# Cinobufagin alleviates lipopolysaccharide-induced acute lung injury by regulating autophagy through activation of the p53/mTOR pathway

**DOI:** 10.3389/fphar.2022.994625

**Published:** 2022-11-28

**Authors:** Cheng Wang, Xianghuang Mei, Yanrong Wu, Yuting Yang, Zhenguo Zeng

**Affiliations:** ^1^ Department of Critical Care Medicine, Medical Center of Anesthesiology and Pain, The First Affiliated Hospital of Nanchang University, Nanchang, China; ^2^ Jiangxi Institute of Respiratory Disease, Nanchang, China; ^3^ Department of Gastrointestinal Surgery, Heji Hospital Affiliated to Changzhi Medical College, Changzhi, China; ^4^ Department of Ophthalmology, Affiliated Hospital of Jiangxi University of Traditional Chinese Medicine, Nanchang, China

**Keywords:** acute lung injury, cinobufagin, lipopolysaccharide, autophagy, p53, mTOR

## Abstract

Acute lung injury (ALI) is a severe clinical disorder characterized by dysregulated inflammatory responses, leading to high rates of morbidity and mortality. Cinobufagin, a primary component isolated from cinobufotalin, exerts strong anticancer effects. However, there are few reports on its role in ALI, and it is unclear whether cinobufagin affects lipopolysaccharide (LPS)-induced ALI. Therefore, the present study aimed to investigate the effect of cinobufagin on LPS-induced ALI and to assess its potential mechanism of action. The results showed that cinobufagin alleviated lung histopathological changes and protected the permeability of lung tissues in LPS-induced ALI. In addition, cinobufagin effectively suppressed inflammatory responses through the induction of autophagy in LPS-induced ALI cells and in a mouse model. Moreover, cinobufagin enhanced autophagy through the p53/mTOR pathway in LPS-induced ALI. Herein, it was reported for the first time that cinobufagin inhibited the inflammatory response of LPS-induced ALI, which laid the foundation for further understanding and development of cinobufagin as a potential new drug for ALI.

## 1 Introduction

Acute lung injury (ALI) is a severe clinical disorder with a high morbidity and mortality rate. Pathological characteristics include rapid alveolar injury, severe hypoxemia, and non-cardiogenic pulmonary edema, which may develop into acute respiratory distress syndrome (ARDS) (Thompson et al., 2017). Bacterial infections are the primary cause of ALI (Kumar, 2020). Lipopolysaccharide (LPS), the main component of the outer cell wall of Gram-negative bacteria, is a key factor in the development of ALI/ARDS (Chaudhuri et al., 2007). Exposure to LPS can lead to the infiltration of inflammatory cells and the generation of cytokines and chemokines, which subsequently results in the initiation and propagation of an inflammatory response in ALI/ARDS (Cheng et al., 2018). Therefore, LPS has been widely used as a model for experimental ALI (Chen et al., 2010). Current ALI treatment strategies include reducing bacterial count and ameliorating organ damage caused by excessive inflammation. Despite improvements in intensive care and mechanical ventilation regimens, the mortality rates as a consequence of pneumonia or sepsis associated with ALI remain high (Butt et al., 2016). Therefore, investigating new and effective means of treating ALI is crucial for improving the health and survival of patients.

Autophagy is a major catabolic process that transports proteins, cytoplasmic components, and organelles to lysosomes for degradation or recycling (Deretic, 2021). The main autophagy-related proteins are LC3-II, Beclin-1, and ATG5 (Dikic and Elazar, 2018) and the key signaling pathways regulating autophagy include the p53/mechanistic target of rapamycin complex 1 (mTORC1) and B-cell lymphoma 2 (Bcl-2) protein families (Maiuri et al., 2010; Houston, 2019). Autophagy is an early adaptive mechanism by which tissues clear organelles or proteins to maintain cell homeostasis. Excessive autophagy results in cell death (Barth et al., 2010). While autophagy plays a vital role in LPS-induced ALI, its specific mechanism remains unclear.

Cinobufotalin is a monomer from Bufo bufo gargarizans Cantor and Bufo melanostictus Schneider extracts used in traditional Chinese medicine (Zhang et al., 2020). Cinobufagin, a primary component isolated from cinobufotalin, has been shown to exert antitumor effects on a number of malignant tumors such as hepatocellular carcinoma, colorectal cancer, lung cancer, and osteosarcoma (Qi et al., 2011; Dai et al., 2018; Bai et al., 2021; Zhang et al., 2022). A previous study showed that cinobufagin attenuated bleomycin-induced pulmonary fibrosis in mice (Li et al., 2019). However, the effect of cinobufagin on LPS-induced ALI remains unclear. Therefore, the aim of the present study was to clarify whether cinobufagin exerts anti-inflammatory effects in LPS-induced ALI. We showed that cinobufagin could induce autophagy to inhibit the production of inflammatory cytokines, such as TNF-α, IL-1β, and IL-6, in LPS-induced ALI, and the p53/mTOR pathway could be involved in cinobufagin-induced autophagy.

## 2 Materials and methods

### 2.1 Materials

Cinobufagin was purchased from the X-Y Biotechnology Company (Shanghai, China). Dimethyl sulfoxide (DMSO) and LPS were purchased from Sigma-Aldrich (St. Louis, MO, United States). Cinobufagin was dissolved in dimethyl sulfoxide to a final concentration of 100 mmol/L. Pifithrin-u was purchased from Selleckchem (Houston, United States). Cell Counting Kit-8 (CCK-8) was purchased from Tongren Institute of Chemistry (Dalian, China); Annexin V-FITC was purchased from BD Biosciences; and ELISA test kits for IL-1B, IL-6, and TNF-a were purchased from Thermo Fisher Scientific (Waltham, MA, United States). TRIzol reagent was obtained from Invitrogen (Carlsbad, CA). Prime Script RT Master Mix and TB Green Premix Ex Taq were purchased from Takara Bio Inc. (Dalian, China). A BCA protein assay kit was purchased from Tiangen Biotech (Beijing, China). RIPA buffer was purchased from Beyotime (Shanghai, China). Primary antibodies against LC3B, Beclin-1, p53, phospho-p53, mTOR, phospho-mTOR, and HRP-linked anti-rabbit IgG were purchased from Cell Signaling Technology (Beverly, MA, United States). Antibody against GAPDH was purchased from Proteintech (Wuhan, China).

### 2.2 Experimental animals and protocols

Animal experimental procedures were approved by the Animal Care and Use Committee of the First Hospital of Nanchang University. C57/BL mice were obtained from SJA Laboratory Animal Company (Hunan, China), housed in a specific pathogen-free (SPF) facility with a light/dark cycle of 12 h/12 h, and randomly assigned to four groups: control, LPS (5 mg/kg), LPS cinobufagin (7.5 mg/kg), and 3–MA (15 mg/kg) LPS + cinobufagin (*n* = 6 per group). An ALI model was established by intratracheal administration of LPS. 3-Methyladenine (3-MA) was administered 1 h before cinobufagin intervention, and cinobufagin was administered 1 h before LPS treatment. The control group was administered equal amounts of PBS simultaneously. Two days after LPS injection, mice were sacrificed for subsequent assays.

### 2.3 Cell culture

Human bronchial epithelial (HBE) cells were purchased from American Type Culture Collection (CRL-2741) and cultured in RPMI 1640 (Gibco, United States) containing 10% fetal bovine serum (FBS, Gibco, United States) in a 5% CO2 incubator at 37°C. Cells were digested with 0.25% trypsin and passaged according to the experimental requirements. Cells were pretreated with 3-MA (1 μM) or cinobufagin (2 μM) for 2 h. Cells were then incubated with LPS (100 μg/ml) for 24 h and harvested for further analysis.

### 2.4 Cell viability assay

Cell viability in the various treatment groups was assessed using a CCK8 assay. HBE cells in the logarithmic phase were inoculated into 96-well plates at a density of 8 × 103 cells/well overnight. Next, culture medium containing 10 ml of CCK-8 solution was added to each well. After incubation for 2 h, the absorbance (optical density value) of each well at a wavelength of 450 nm was measured using a microplate reader (Waltham, MA, United States).

### 2.5 Apoptosis analysis

HBE cells were seeded in six-well plates at a density of 10 × 10^4^ cells/well. Following incubation with 3-MA (1 μM) or cinobufagin (2 μM) for 2 h, HBE cells were treated with LPS (100 μg/mg) in complete medium for 24 h. Cells were digested with trypsin and washed twice with pre-cooled PBS. Next, the cells were resuspended in 100 μL binding buffer, mixed with 5 μL annexin V-FITC and 5 μL PI, and incubated for 15 min in the dark. Following the addition of 400 μL binding buffer, apoptotic cells were detected by flow cytometry.

### 2.6 ELISA

The protein levels of IL-1β, IL-6, and TNF-α in the bronchoalveolar lavage fluid (BALF) and cell supernatant were detected using an ELISA kit according to the manufacturer’s instructions.

### 2.7 RNA isolation and quantitative real-time PCR (qPCR)

Total RNA was extracted from the cells using TRIzol reagent. Reverse transcription was performed with total RNA (1,000 ng) using SYBR®Premix Ex Taq™ (TaKaRa, Shiga, Japan). qRT-PCR was performed using TB Green Premix Ex Taq (TaKaRa, Shiga, Japan) following the manufacturer’s instructions. Each sample was assayed in triplicate. The ΔΔCt method was used to quantify and compare mRNA levels to GAPDH. The primers used are listed in Table 1.

**TABLE 1:** Primers for quantitative real-time PCR.

Gene	Sense (5′–3′)	Anti-sense (5′–3′)
GAPDH	CAT​TGG​CTA​CGA​ATA​CAG​CA	AGG​GGC​AAC​TGG​TCT​ACA​TG
IL-1β	TCA​TTG​TGG​CTG​TGG​AGA​AG	AGG​CCA​CAG​GTA​TTT​TGT​CG
IL-6	ACT​CAC​CTC​TTC​AGA​ACG​AAT​TG	CCA​TCT​TTG​GAA​GGT​TCA​GGT​TG
TNF-α	CCT​CTC​TCT​AAT​CAG​CCC​TCT​G	GAG​GAC​CTG​GGA​GTA​GAT​GAG

### 2.8 Western blot analysis

Protein concentration was determined using a BCA protein assay kit. A total of 20 μg of protein was obtained from each group and separated using SDS-PAGE. After electrophoresis, the proteins were electrotransferred to nitrocellulose membranes and blocked with 5% skimmed milk at room temperature for 1 h. Different primary antibodies were added according to the experimental requirements and incubated overnight at 4°C. After washing with TBS-Tween-20 (TBS-T), the membranes were incubated with secondary antibody at room temperature for 1 h, and subsequently washed with TBS-T. The protein bands were visualized using SH-Cute 523 (Hangzhou, China).

### 2.9 Lung pathology

Lung tissues were fixed in a 4% paraformaldehyde solution for more than 2 days. Thereafter, the tissues were dehydrated in water through alcohol and embedded in paraffin. A series of microsections were cut using a microtome and stained with hematoxylin and eosin (HE). The extent of lung injury was scored by two independent pathologists.

### 2.10 Wet/dry (W/D) weight ratio

Following treatment of mice with LPS, cinobufagin, and 3-MA at different concentrations, fresh lung tissues were weighed to obtain the wet weight. The lung tissues were then dried for 48 h and weighed to determine their dry weight. Subsequently, the lung W/D ratio was calculated. Each experiment was repeated at least thrice.

### 2.11 Total cell and protein concentration in the bronchoalveolar lavage fluid

Bronchoalveolar lavage fluid was collected by intratracheal injection of 1 ml of pre-cold PBS and centrifuged at 1,000 rpm for 10 min. After red blood cells were lysed, the remaining cells were washed, resuspended in PBS, counted, and the protein concentration in the supernatant was assessed using a BCA protein assay kit.

### 2.12 Immunofluorescence staining

HBE cells were fixed with 4% paraformaldehyde. The cells were permeabilized with 0.3% Triton X-100, blocked with 1% bovine serum albumin (BSA), and incubated with antibodies against LC3B (1:500) overnight at 4°C. After washing with PBS thrice, the cells were incubated with secondary antibodies for 1 h. Next, the cell nuclei were stained with 4’6’-diamino-2-phenylindole (DAPI) and fluorescence images were captured using a confocal laser scanning microscope (Leica, Germany).

### 2.13 Statistical analysis

Unless otherwise stated, data are presented as the mean standard error of one representative of three independent experiments, each of which was conducted in triplicate. The Student’s t-test was performed to evaluate the differences between the two groups. Qualitative variables were compared using the chi-square (χ2) test, and *p* < 0.05 was considered to be statistically significant (*p < 0.05; ** *p* < 0.01; *** *p* < 0.001). Graphics were generated using the GraphPad Prism 8.1.1 software. All statistical analyses were performed using SPSS software (IBM SPSS Statistics 21.0).

## 3 Results

### 3.1 Cinobufagin alleviates lipopolysaccharide-induced lung histopathological changes and protects lung tissue permeability in lipopolysaccharide-induced acute lung injury

**FIGURE 1:**
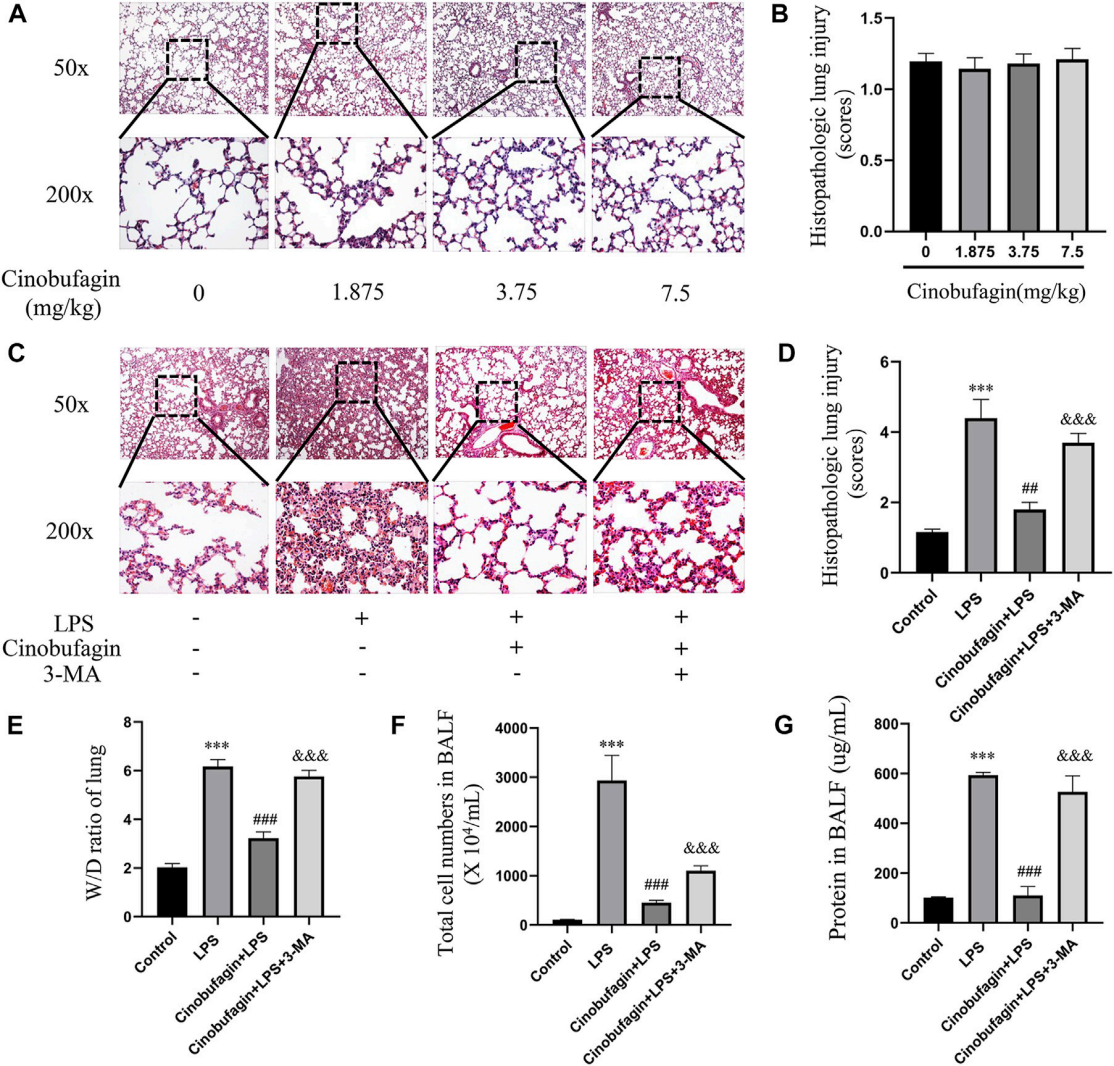
Effects of cinobufagin on LPS-induced histopathologic changes and edema in LPS-induced ALI in the lungs. **(A)** 24 h after different concentrations of cinobufagin injection, the lungs in each group were prepared for histological evaluation. Representative histological section of the lungs was stained by HE staining, magnification (50x, bar = 100 μm; 200x, bar = 50 μm). **(B)** The lung injury scores were determined. **(C)** 48 h after LPS injection, the lungs in each group were prepared for histological evaluation. **(D)** The lung injury scores were determined. **(E)** Lung tissues were weighed to calculate the W/D ratio. **(F)** The total cells detected by flow cytometry in BALF. **(G)** BCA assay was used to determine the total protein concentration in BALF. The values presented are mean ± SEM, ***p < 0.001 compared with control group. ##*p* < 0.01, ###*p* < 0.001 in comparison to LPS group. &&&*p* < 0.001 versus the cinobufagin + LPS group.

Lung injury in mice was assessed by HE staining of lung tissue 48 h after intratracheal LPS injection. First, we separately explored the effects of different concentrations of cinobufagin (1.875, 3.75 mg/kg and 7.5 mg/kg) on mice and found that none of them caused significant inflammatory changes compared to the control group (Figures 1A,B). Therefore, 7.5 mg/kg was selected for subsequent experiments. In addition, as shown in Figures 1 C–D, the control group showed normal lung tissue structure. In the LPS-induced group, lung tissue was severely damaged with a destroyed alveolar structure, thickened pulmonary septum, and edematous and inflammatory cells infiltrated in the alveoli and interstitium. Pathological processes were ameliorated by cinobufagin (7.5 mg/kg) in mice, and these phenomena were reversed by 3-MA treatment.

In ALI, when the epithelial or endothelial cell barrier is damaged, increased permeation of macromolecules and fluids into the interstitium is noted (Johnson and Matthay, 2010). The wet-to-dry weight ratio of the lungs, total cells, and BALF protein concentration were markers used to assess edema in ALI. Forty-8 h after LPS injection, the wet-to-dry weight ratio of the lung tissue, total cells, and BALF protein concentration increased. However, cinobufagin (7.5 mg/kg) blocked this increase, and the inhibitory effects of cinobufagin were reversed by 3-MA (Figures 1E–G). Taken together, the data shows that cinobufagin relieves the severity of increased lung permeability in LPS-induced ALI.

### 3.2 Cinobufagin suppresses inflammatory responses through induction of autophagy in lipopolysaccharide-induced acute lung injury mouse model

**FIGURE 2:**
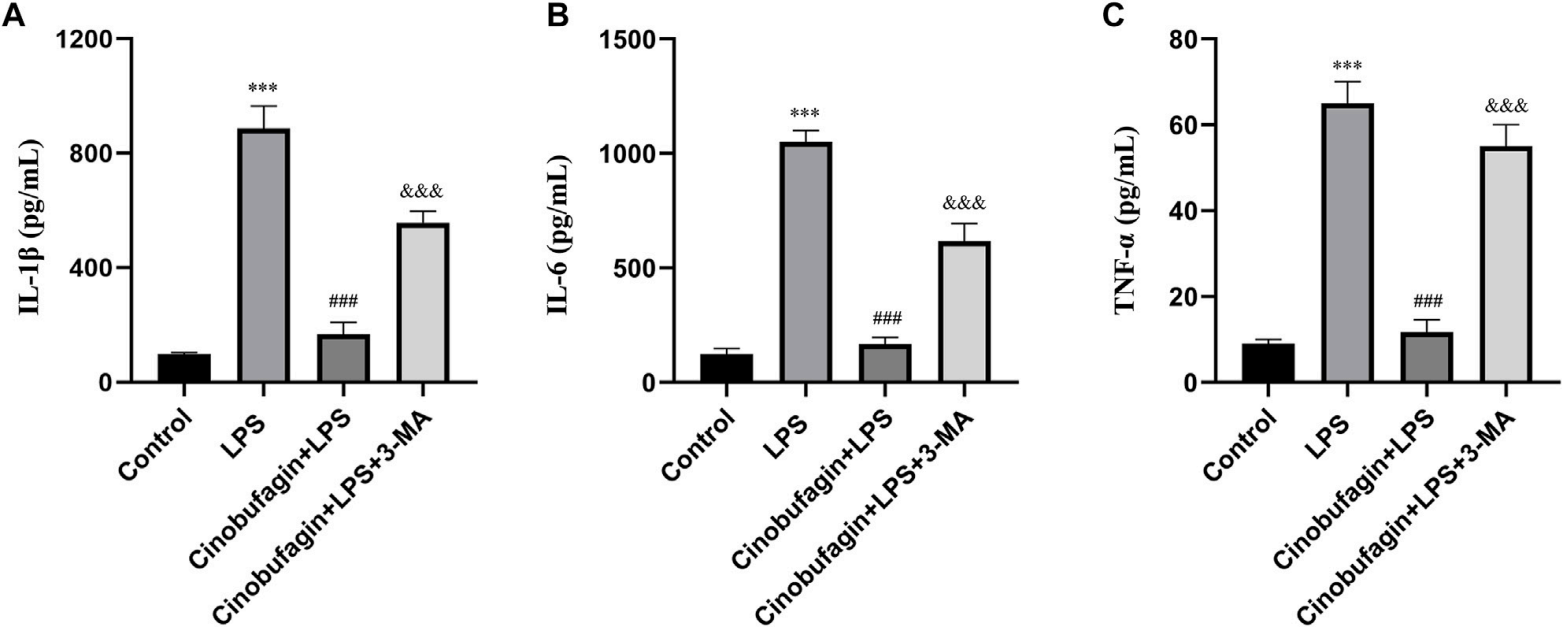
Cinobufagin inhibits lung inflammatory injury through autophagy activation *in vivo*. **(A)** IL-1β in BALF 48 h after LPS-induced ALI. **(B)** IL-6 in BALF 48 h after LPS-induced ALI. **(C)** TNF-α in BALF 48 h after LPS-induced ALI. The values presented are mean ± SEM, ***p < 0.001 compared with control group. ###*p* < 0.001 in comparison to LPS group. &&&*p* < 0.001 versus the cinobufagin + LPS group.

Inflammatory cytokines play key roles in LPS-induced ALI. We measured the levels of IL-1β, IL-6, and TNF-α in BALF to assess the severity of inflammatory responses in the lungs using ELISA. As shown in Figures 2A–C, the levels of IL-1β, IL-6, and TNF-α in BALF were significantly elevated in the LPS group compared to the control group. However, the cinobufagin-treated group in LPS-induced ALI showed significantly lower levels of IL-1β, IL-6, and TNF-α. Additionally, the inhibitory effects of cinobufagin were reversed by 3-MA. Thus, these results show that cinobufagin decreases LPS-induced lung inflammatory responses through the activation of autophagy *in vivo*.

### 3.3 Cinobufagin enhanced autophagy through the p53/mTOR pathway in an lipopolysaccharide-induced acute lung injury mouse model

**FIGURE 3:**
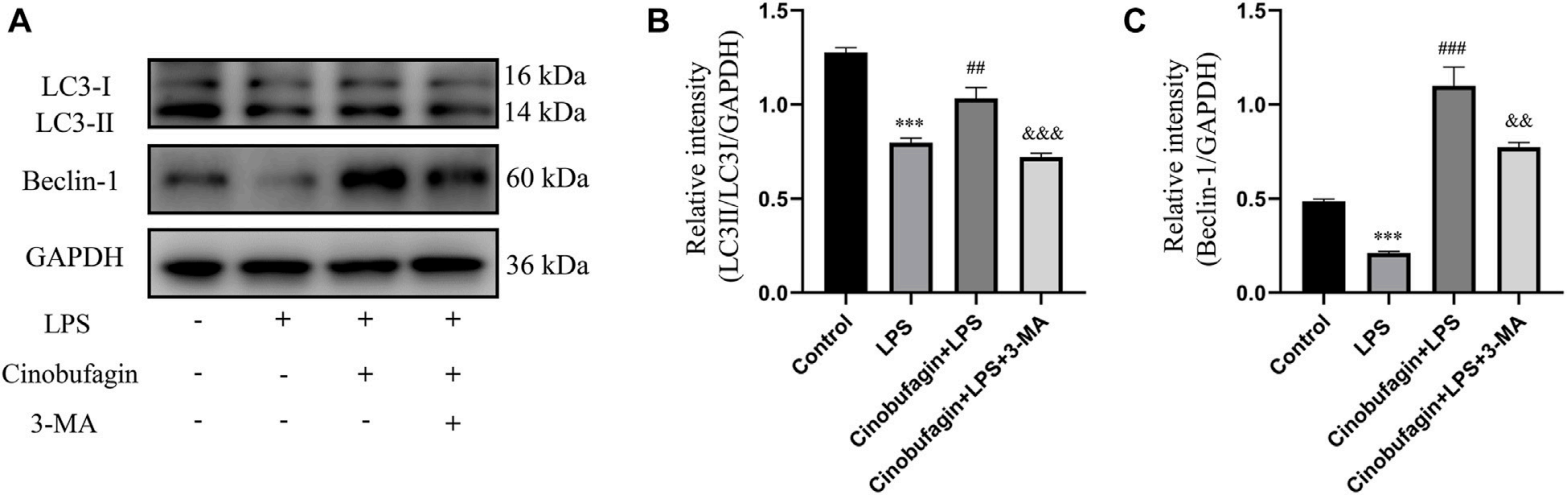
Cinobufagin induces autophagy *in vivo*. **(A)** Western blot detected the levels of LC3-II/I and Beclin-1 in lung tissues. **(B–C)** Quantitative analysis of LC3-II/I and Beclin-1 were shown in bar graphs, respectively. The values presented are mean ± SEM, ***p < 0.001 compared with control group. ##*p* < 0.01, ###*p* < 0.001 in comparison to LPS group. &&*p* < 0.01, &&&*p* < 0.001 versus the cinobufagin + LPS group.

To elucidate the effect of cinobufagin on autophagy in LPS-induced lung injury, we first evaluated the protein levels of LC3-II/I and Beclin-1 using western blotting. As shown in Figures 3A–C, the expression of LC3-II/I and Beclin-1 was significantly decreased in the LPS group. Moreover, pretreatment with cinobufagin further enhanced the levels of LC3-II/I and Beclin-1 in an LPS-induced ALI mouse model. Importantly, autophagy activation induced by cinobufagin was reversed by 3-MA *in vivo*. Our results indicate that cinobufagin enhances autophagy in an LPS-induced ALI mouse model.

**FIGURE 4:**
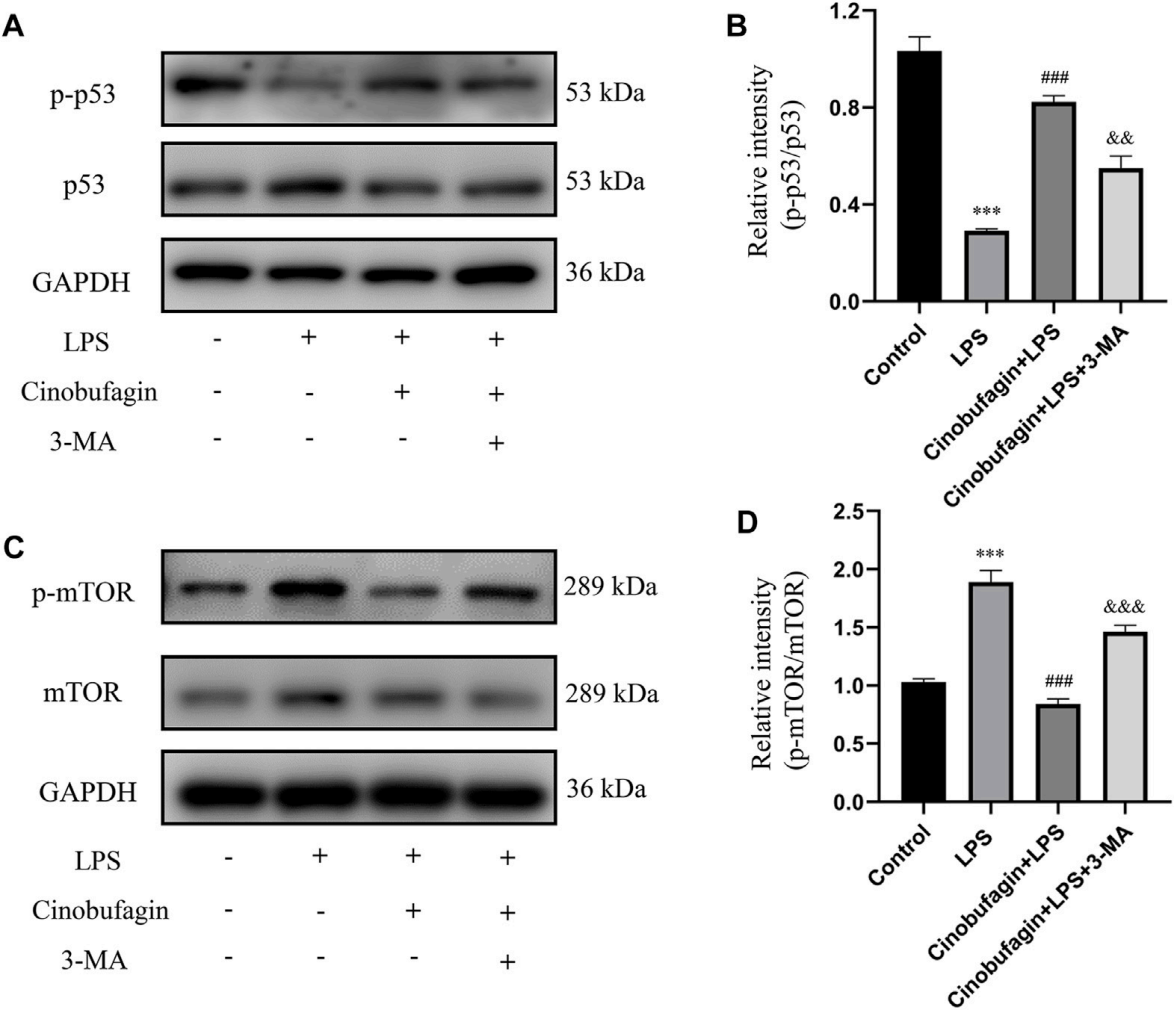
Cinobufagin induces autophagy through regulating the p53/mTOR pathway *in vivo*. **(A–B)** The levels of p-p53 and p53, and **(C, D)** p-mTOR and mTOR were evaluated by Western blot. The values presented are mean ± SEM, ***p < 0.001 compared with control group. ##*p* < 0.01, ###*p* < 0.001 in comparison to LPS group. &&*p* < 0.01, &&&*p* < 0.001 versus the cinobufagin + LPS group.

To further identify the mechanisms of autophagy activated by cinobufagin in LPS-induced ALI *in vivo*, we investigated the p53/mTOR pathway. Cinobufagin treatment (7.5 mg/kg) resulted in increased levels of p-p53 and decreased levels of p-mTOR proteins. While these effects were reversed by 3-MA treatment, total p53 and mTOR levels did not change significantly in the LPS-induced ALI mouse model (Figures 4A–D). These results suggest that the p53/mTOR pathway governs cinobufagin-induced enhancement of autophagy in ALI *in vivo*.

### 3.4 Cinobufagin promotes cell viability and protects against lipopolysaccharide-induced apoptosis in human bronchial epithelial cells

**FIGURE 5:**
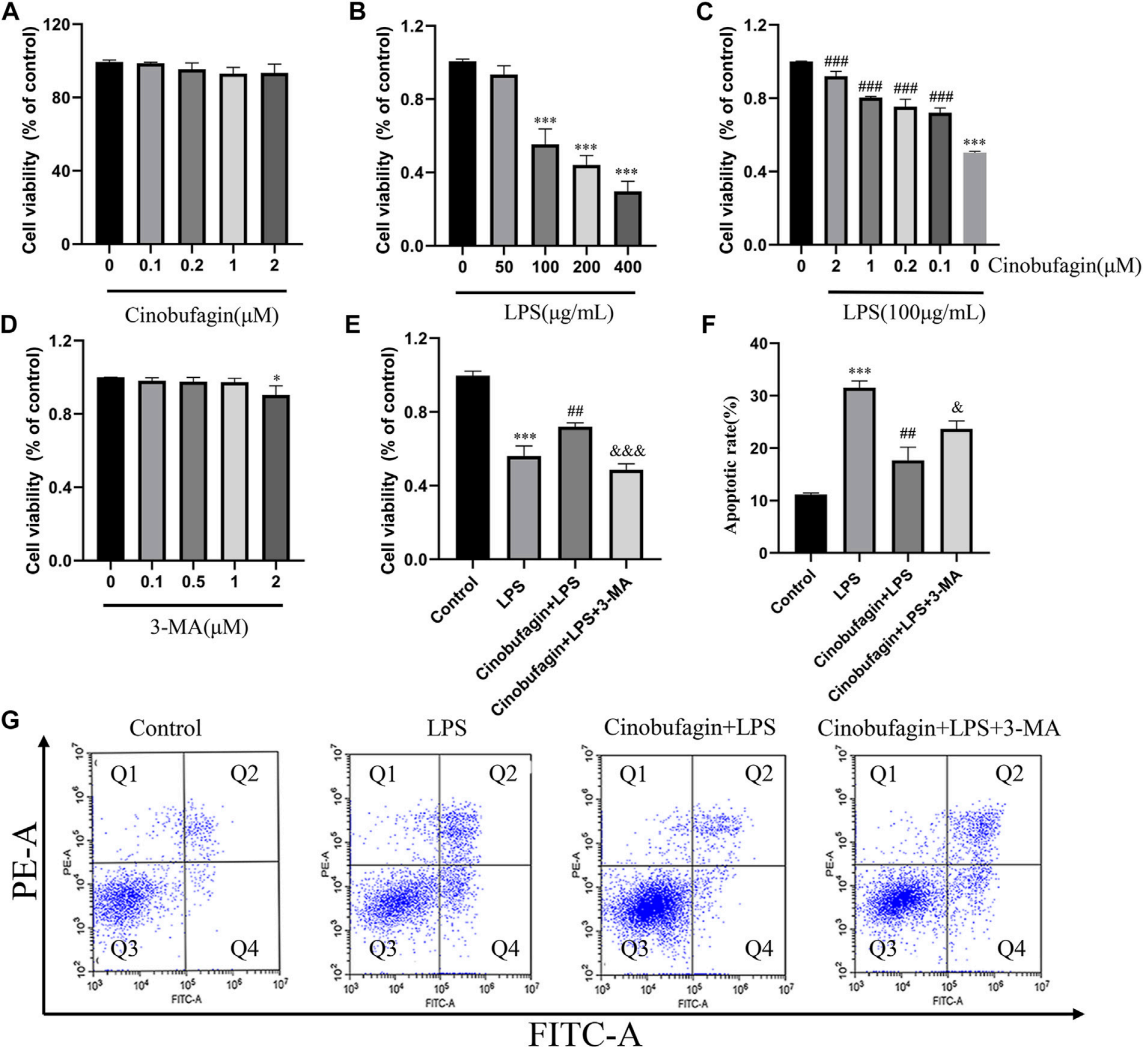
Cinobufagin promotes cell viability and protects LPS-induced apoptosis in HBE cells. **(A–E)** Viability of HBE cells was estimated using the CCK8 assay. **(F)** Quantitative analysis of cell apoptosis in HBE cells. **(G)** Apoptosis of HBE cells was stained with Annexin-VFITC and PI and analyzed by flow cytometry. The values presented are mean ± SEM, ***p < 0.001 compared with control group. ##*p* < 0.01, ###*p* < 0.001 in comparison to LPS group. &< 0.05, &&*p* < 0.01, &&&*p* < 0.001 versus the cinobufagin + LPS group.

The epithelium is considered the primary defender of the airway and alveolus and forms a strong natural barrier against infectious and non-infectious stimuli in the pathogenesis and treatment of ALI (Crosby and Waters, 2010). Therefore, we used HBE cells for subsequent experiments. The effect of cinobufagin on the viability of HBE cells was assessed using the CCK8 assay. Cinobufagin demonstrated no significant cytotoxic effect on HBE cell viability (Figure 5A). To determine the working concentration of LPS, HBE cells were treated with varying concentrations of LPS (0, 50, 100, 200, and 400 μg/ml) for 24 h, and cell viability was assessed. LPS decreased the viability of HBE cells in a dose-dependent manner (Figure 5B). Based on these results, we used 100 μg/ml LPS for subsequent experiments. Next, we determined whether cinobufagin exerts a protective effect on HBE cells exposed to LPS. We pre-treated HBE cells with varying concentrations of cinobufagin (2 h) prior to LPS (100 μg/ml) exposure for 24 h, in which cinobufagin increased the decline in cell viability as a result of LPS challenge (Figure 5C). Thereafter, 3-MA, an autophagy inhibitor, was used to detect whether autophagy inhibition could decrease the viability of HBE cells. Results indicated that 3-MA (2 μM) reduced the viability of the cells (Figure 5D). Based on these results, we employed 2 μM cinobufagin and 1 μM 3-MA in subsequent experiments. To determine whether cinobufagin could increase the viability of HBE cells and whether 3-MA reversed the protective effect of cinobufagin, the cells were incubated with 100 μg/ml LPS, 2 μM cinobufagin, and/or 1 μM 3-MA. Cell viability was significantly increased with pre-treatment of cinobufagin and reversed by 3-MA (Figure 5E). To assess whether cinobufagin could increase the protective action against the cytotoxic effects of LPS and whether 3-MA reversed the protective effect, we pre-treated HBE cells with cinobufagin or 3-MA (2 h), followed by incubation with LPS (100 μg/ml) for 24 h. Apoptosis was assessed by PI and Annexin V-FITC double staining using flow cytometry. As shown in Figures 5 F–G, the rate of apoptosis was significantly increased by pretreatment with cinobufagin and reversed by 3-MA and cinobufagin treatment simultaneously in LPS-induced HBE cells.

### 3.5 Cinobufagin suppresses lipopolysaccharide-induced proinflammatory factor expression in human bronchial epithelial cells

**FIGURE 6:**
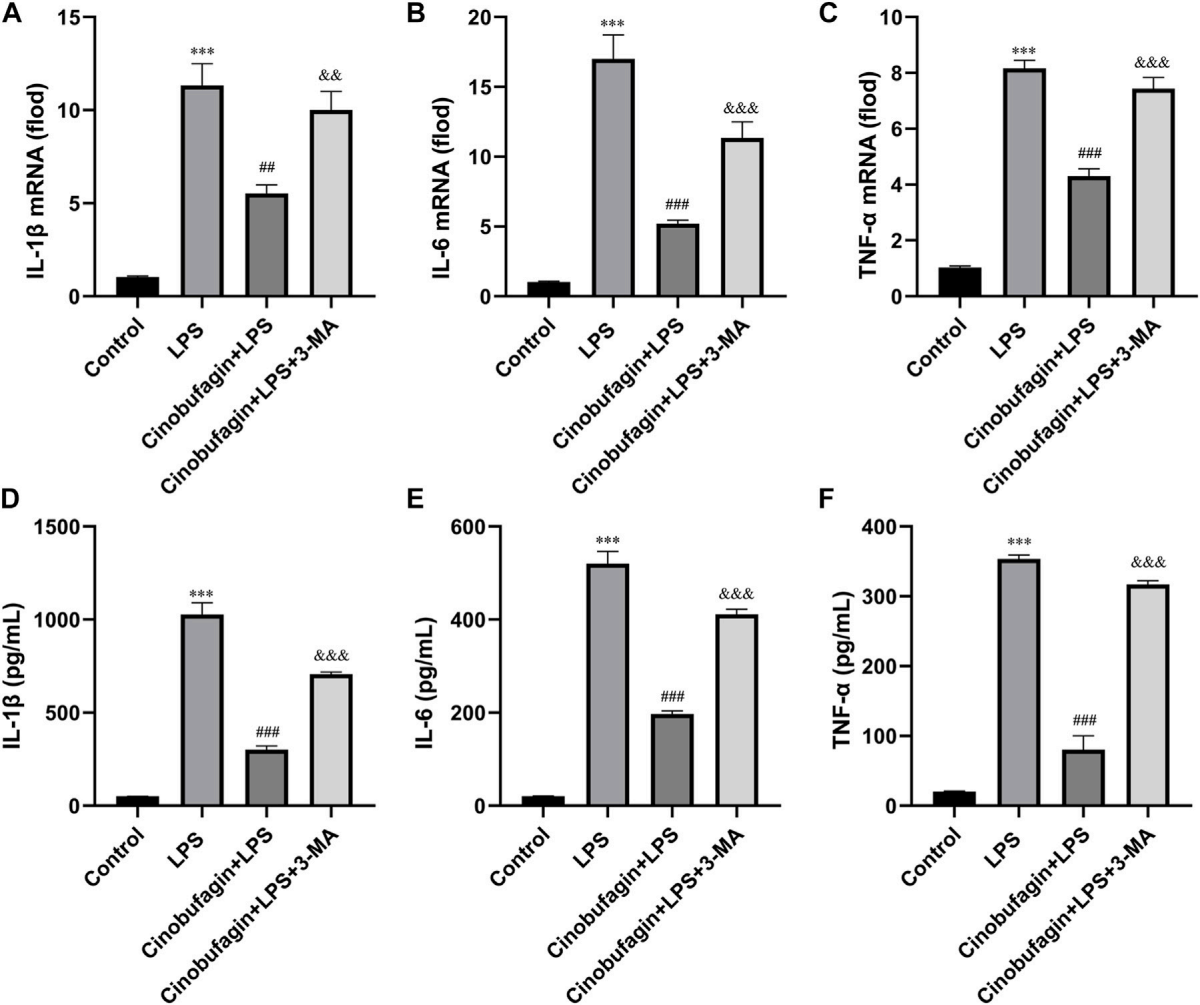
Cinobufagin decreased proinflammatory cytokine production in LPS-induced HBE cells. HBE cells were pre-treated with 2 μM cinobufagin or 1 μM 3-MA for 2 h before LPS (100 μg/ml) for 24 h, cells were collected to isolate total RNA, the mRNA expression level of **(A)** IL-1β, **(B)** IL-6, **(C)** TNF-α were measured by quantitative PCR. The secretion of **(D)** IL-1β, **(E)** IL-6, and **(F)** TNF-α in the supernatants was assayed by ELISA. The values presented are mean ± SEM, ***p < 0.001 compared with control group. ##*p* < 0.01, ###*p* < 0.001 in comparison to LPS group. &&*p* < 0.01, &&&*p* < 0.001 versus the cinobufagin + LPS group.

The lung epithelium is essential for LPS-induced inflammatory responses (Gropper and Wiener-Kronish, 2008). We examined whether cinobufagin could affect the production of inflammatory cytokines in HBE cells using quantitative RT-PCR. As shown in Figures 6A–C, compared with the LPS group, cinobufagin signficantly suppressed the production of the proinflammatory cytokines, IL-1β, IL-6 and TNF-α; however, the inhibitory effects of cinobufagin were reversed by 3-MA in HBE cells treated with LPS. Similarly, cytokine secretion in HBE cells was detected by ELISA, which showed that the levels of IL-1β, IL-6, and TNF-α were also markedly attenuated by cinobufagin treatment. However, the inhibitory effects of cinobufagin were reversed by 3-MA (Figures 6D–F).

### 3.6 Cinobufagin induces autophagy through the p53/mTOR pathway in human bronchial epithelial cells with lipopolysaccharide treatment

**FIGURE 7:**
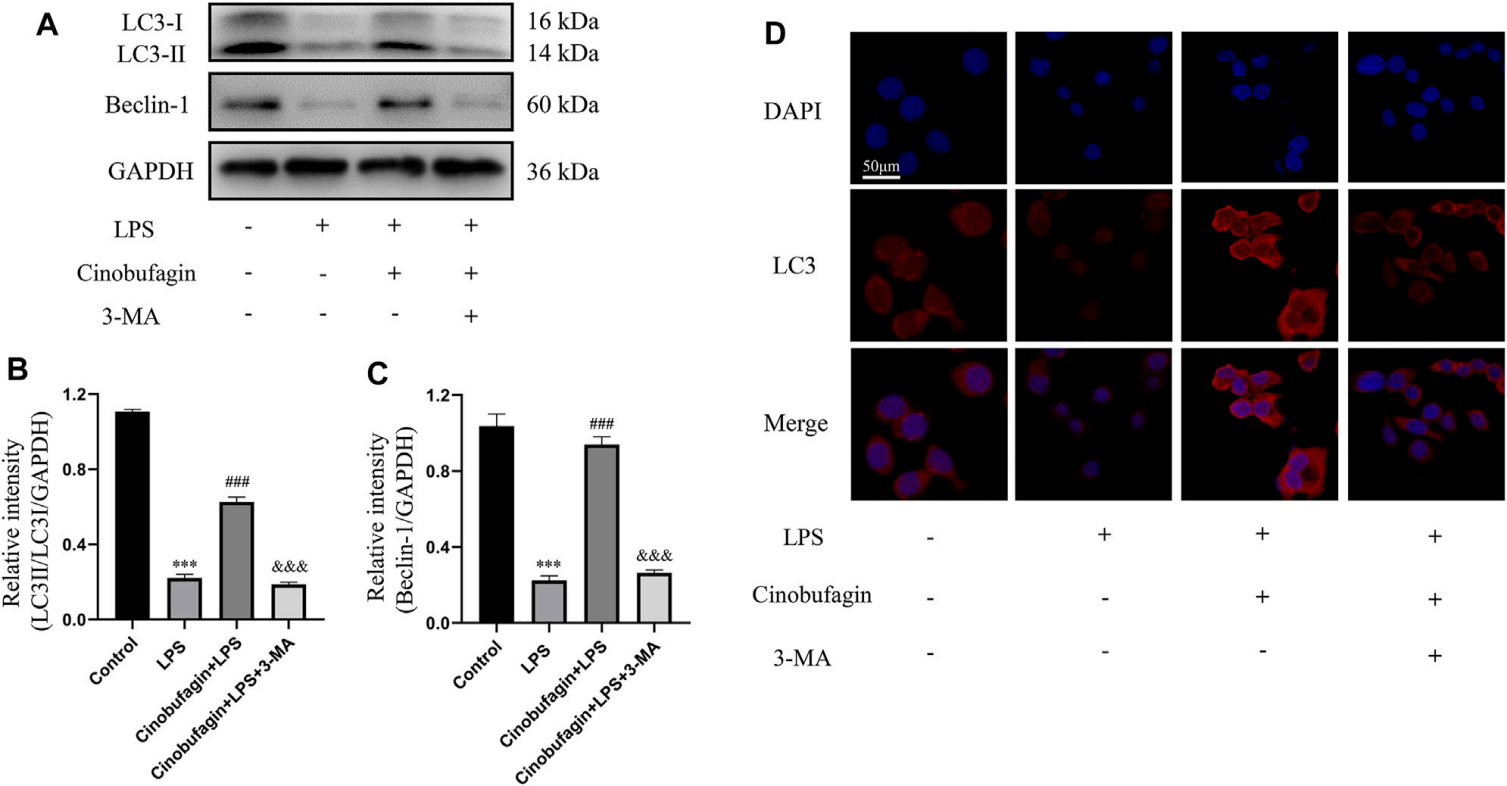
Cinobufagin induces autophagy in LPS-induced HBE cells. **(A–C)** The levels of LC3-II/I and Beclin-1 were detected by Western blot in HBE cells, and the bar graphs showed the quantitative analysis results of the corresponding protein in each group, respectively. **(D)** The representative images of the fluorescence of LC3 in HBE cells (magnification ×50). The values presented are mean ± SEM, ***p < 0.001 compared with control group. ##*p* < 0.01, ###*p* < 0.001 in comparison to LPS group. &&*p* < 0.01, &&&*p* < 0.001 versus the cinobufagin + LPS group.

To investigate whether the protective effect of cinobufagin on HBE cells was associated with autophagy activation, we measured the levels of several key autophagy-related proteins by western blotting. Treatment with LPS significantly downregulated the ratio of LC3-II/LC3-I and the level of Beclin-1, suggesting the suppression of autophagy (Figures 7A–C). However, cinobufagin activated autophagy, and 3-MA reversed this effect in LPS-induced HBE cells. Subsequently, pre-treatment with cinobufagin obviously upregulated the fluorescence intensity of LC3 observed under CLSM (Figure 7D), indicating autophagy activation in the cells treated with LPS. In addition, the fluorescence intensity of LC3 significantly decreased in the 3-MA group. These results indicated that cinobufagin could induce autophagy in ALI *in vitro*.

**FIGURE 8:**
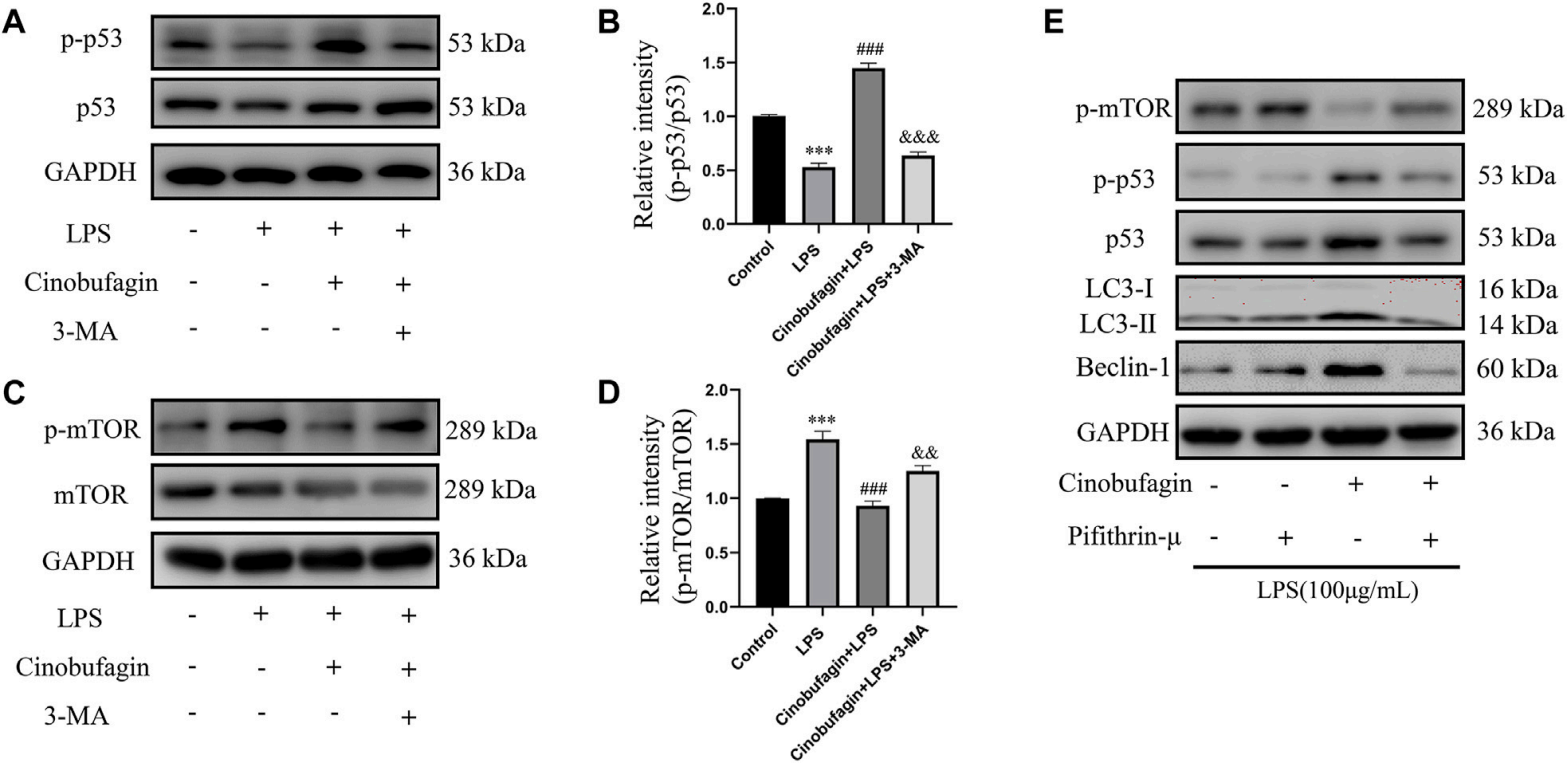
Cinobufagin enhances autophagy through the p53/mTOR pathway *in vitro*. Western blot detected the levels of p-p53, p53, p-mTOR, mTOR, LC3B, Beclin1 **(A–E)**. The values presented are mean ± SEM, ***p < 0.001 compared with control group. ###*p* < 0.001 in comparison to LPS group. &&*p* < 0.01, &&&*p* < 0.001 versus the cinobufagin + LPS group.

The possible mechanisms of autophagy activation by cinobufagin in LPS-induced HBE cells were further investigated. We assessed the p53/mTOR pathway, a classic autophagy-related pathway. Notably, treatment with LPS significantly decreased the phosphorylation of p53 and increased the phosphorylation of mTOR compared to the control group. However, it was observed that cinobufagin activated the p53/mTOR pathway by significantly increasing the phosphorylation of p53 (p-p53) and decreasing the phosphorylation of mTOR (p-mTOR). These effects were reversed by 3-MA (Figures 8A–D). To verify the effect of the p53/mTOR signaling pathway in this study, the p53 inhibitor pifithrin-μ was used to evaluate the protective effect of cinobufagin against LPS-induced ALI. When combining pifithrin-μ with cinobufagin, the proteins p-p53, p53, p-mTOR, Beclin1, and LC3B-II/I were significantly restored (Figure 8E). These results suggest that cinobufagin induced autophagy, at least partly through the regulation of the p53/mTOR pathway in HBE cells treated with LPS.

## 4 Discussion

Acute lung injury is caused by a variety of factors that cause alveolar epithelial cells and capillary endothelial cells to undergo apoptosis and sloughing, triggering lung tissue to develop diffuse edema and other pathological changes. These changes eventually manifest as acute hypoxic respiratory insufficiency and other symptoms (Rubenfeld et al., 2005). ALI is mainly characterized by progressive hypoxemia, respiratory distress, and heterogeneous exudative lesions on lung imaging. If left untreated, ALI can progress to ARDS and involve various tissues and organs, including the heart and kidney (Thompson et al., 2017). Currently, there are no definitive treatments for ALI in clinical practice, however, primary disease treatment and respiratory support is advocated.

With increasing awareness of the molecular mechanisms of disease occurrence and development, the discovery of active pharmaceutical ingredients from natural products for the treatment of various diseases has become a major goal for modern medical workers (Arulselvan et al., 2016; Evans et al., 2020; Atanasov et al., 2021). Cinobufagin, a natural product extracted from toad venom, is one of the major active ingredients in cinobufotalin injections which are used to treat intermediate and advanced tumors. However, the effects of cinobufagin on ALI remain unclear. In this study, we found that cinobufagin increased the viability and decreased the production of TNF-α, IL-1β, and IL-6 in LPS-induced HBE cells. Interestingly, cinobufagin reduced LPS-induced TNF-α to a greater degree than IL-6 and IL-8, which requires further exploration. Cinobufagin inhibited the secretion of inflammatory cytokines in an ALI mouse model. In addition, cinobufagin had little effect on the W/D ratio and BALF protein concentration when compared to the significant decrease in total number of cells. Based on these findings, we further studied the effect of cinobufagin on ALI and attempted to reveal its potential mechanism.

In recent years, autophagy has attracted considerable attention because of its effects on various physiological and pathological processes. Autophagy involves various autophagy-related proteins that engulf and kill pathogens to protect cells and inhibit the secretion of inflammasomes and inflammatory factors (Lo et al., 2013; Shibutani et al., 2015). According to a previous study, autophagy maintains the integrity of the endothelial barrier during LPS-induced lung injury (Zhang et al., 2018). Zhao reported that autophagy activation is involved in the pathophysiological process of sepsis, and alleviates excessive cytokine release and lung injury in sepsis (Zhao et al., 2019). Herein, we found that cinobufagin could activate autophagy in an LPS-induced ALI model. Moreover, cinobufagin significantly increased the protein ratio of LC3-II/LC3-I and the protein level of Beclin-1, as well as upregulated the fluorescence intensity of LC3B. During autophagy, LC3-I is converted to LC3-II, and the ratio of LC3-II/LC3-I is taken as a quantitative index of autophagic activity. Therefore, our study showed that cinobufagin activates autophagy in LPS-induced ALI. Importantly, the activation of autophagy by cinobufagin was reversed by 3-MA treatment. On the basis of these reports and our previous results, we hypothesized that cinobufagin regulates the expression of inflammatory cytokines by regulating autophagy in ALI. After inhibition of autophagy *in vitro* and *in vivo*, the cytokines IL-1β, IL-6, and TNF-α were inhibited. In conclusion, the anti-inflammatory effects of cinobufagin may be related to the induction of autophagy.

**FIGURE 9:**
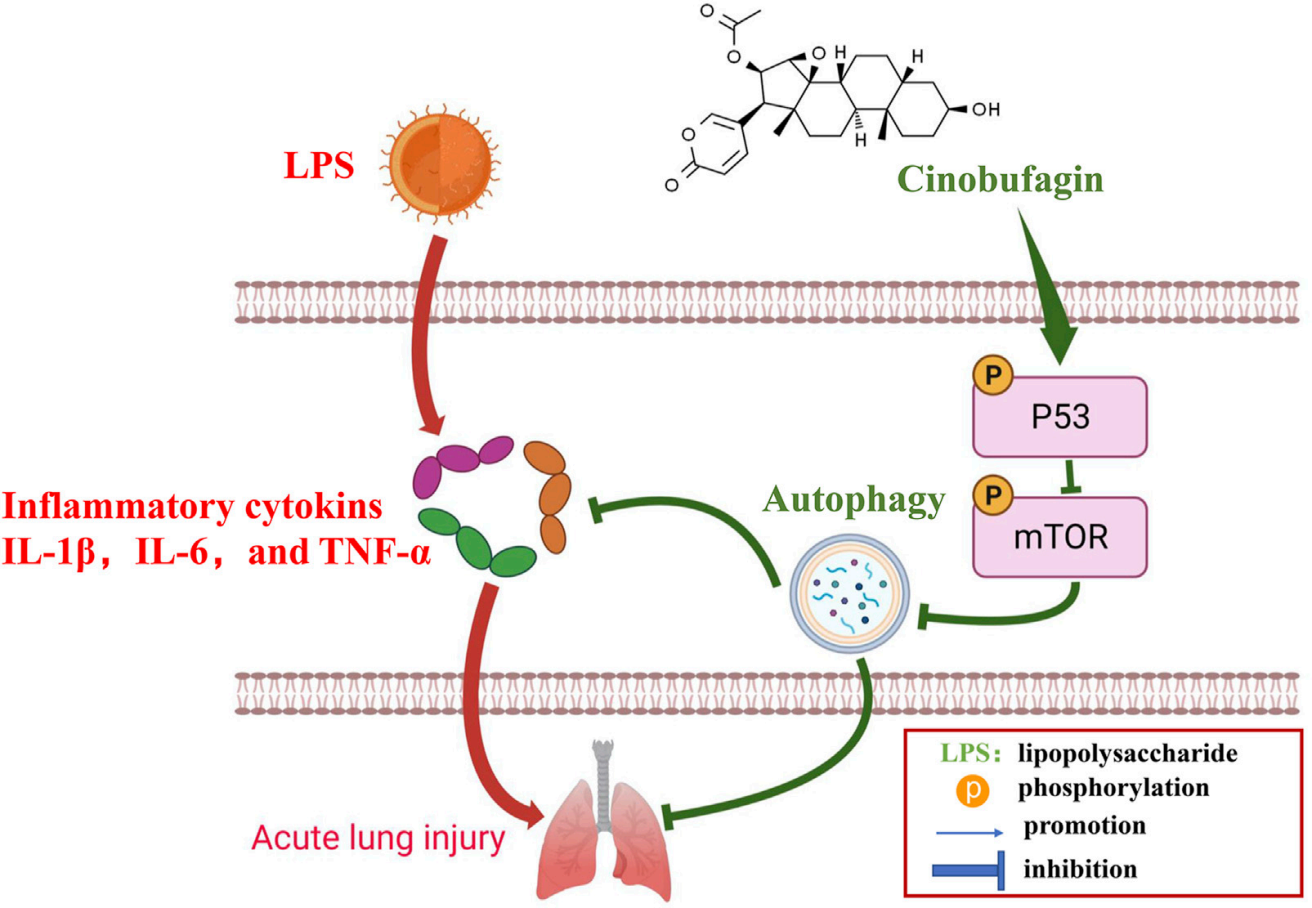
A diagram depicting the mechanism of Cinobufagin ameliorates LPS-induced ALI by activating autophagy through regulating of the p53/mTOR pathway.

Many previous studies have found that the p53 signaling pathway plays a vital role in cell growth, apoptosis, autophagy, and many other cell functions (Chen, 2016; Guo et al., 2016). While p53 protein can activate autophagy, autophagy also inhibits p53 levels and function. This association is functionally intertwined and important for stress response, metabolism, and cancer (White, 2016; Yang et al., 2020). Previous studies have shown that mTOR is a major inhibitor of autophagy (Laplante and Sabatini, 2012). Similarly, it has been reported that activation of mTOR promotes LPS-induced ALI in lung epithelium (Hu et al., 2016). In terms of mechanism, there are multiple pathways involved in the regulation of mTOR, such as p53, MAPK, PI3K-Akt, and AMPK. According to the former study, chitooligosaccharides, could inhibit tumor progression and induce autophagy through the activation of the p53/mTOR pathway in osteosarcoma (Pan et al., 2021). In addition, Uzhytchak et al. found that iron oxide nanoparticle-induced autophagic flux was regulated by the interplay between the p53-mTOR axis in hepatic cells (Uzhytchak et al., 2020). Importantly, our results showed that cinobufagin increased p-p53 protein levels and reduced p-mTOR protein levels in LPS-induced ALI, while total p53 and mTOR showed no evident changes. Pifithrin-μ, a p53 inhibitor, was used to treat the HBE cells. When combining pifithrin-μ with cinobufagin, the proteins p-p53, p53, p-mTOR, Beclin1, and LC3B-II/I were significantly restored, indicating that cinobufagin induced autophagy at least party through the p53/mTOR pathway. Therefore, as shown in Figure 9, cinobufagin improved LPS-induced ALI by regulating the p53/mTOR pathway *via* autophagy activation.

In summary, we demonstrated that cinobufagin effectively suppressed inflammation and alleviated LPS-induced ALI *in vivo* and *in vitro*, at least to a large extent, through regulation of the p53/mTOR pathway *via* autophagy activation. Thus, cinobufagin may be an effective treatment for ALI in clinical practice.

## Data Availability

The original contributions presented in the study are included in the article/Supplementary Materials, further inquiries can be directed to the corresponding author.
