# Supplementary material for: Cinobufagin alleviates lipopolysaccharide-induced acute lung injury by regulating autophagy through activation of the p53/mTOR pathway
Source: Front Pharmacol. 2022 Nov 28;13:994625. doi: 10.3389/fphar.2022.994625 (PMC9742439; doi:10.3389/fphar.2022.994625)

Fig3

LC3B


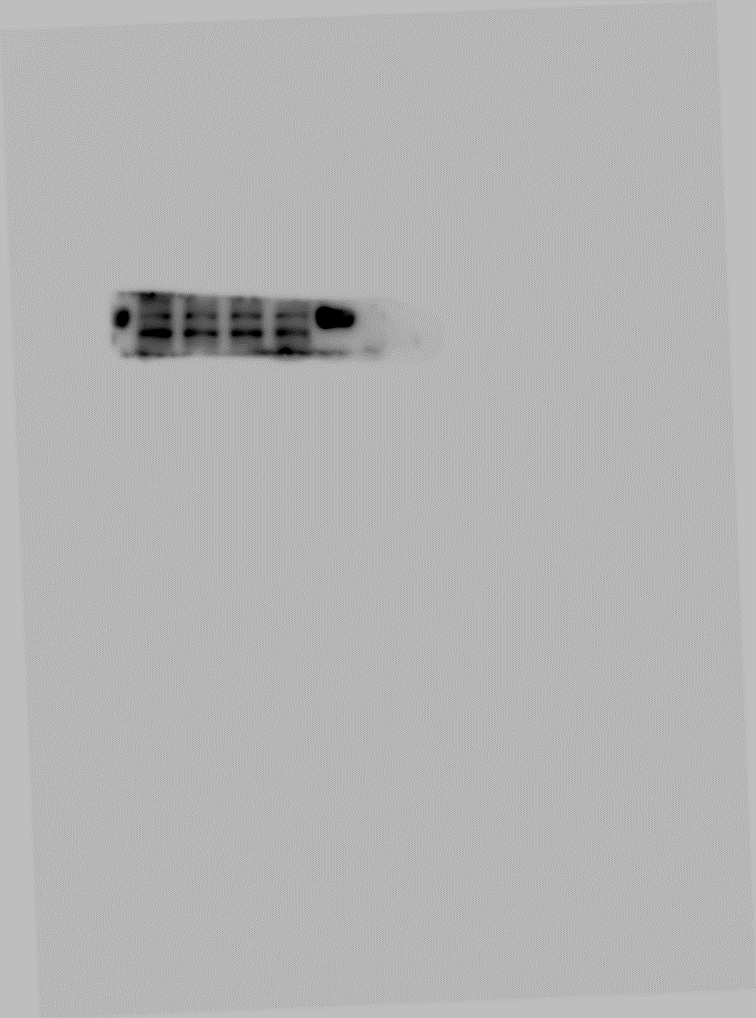


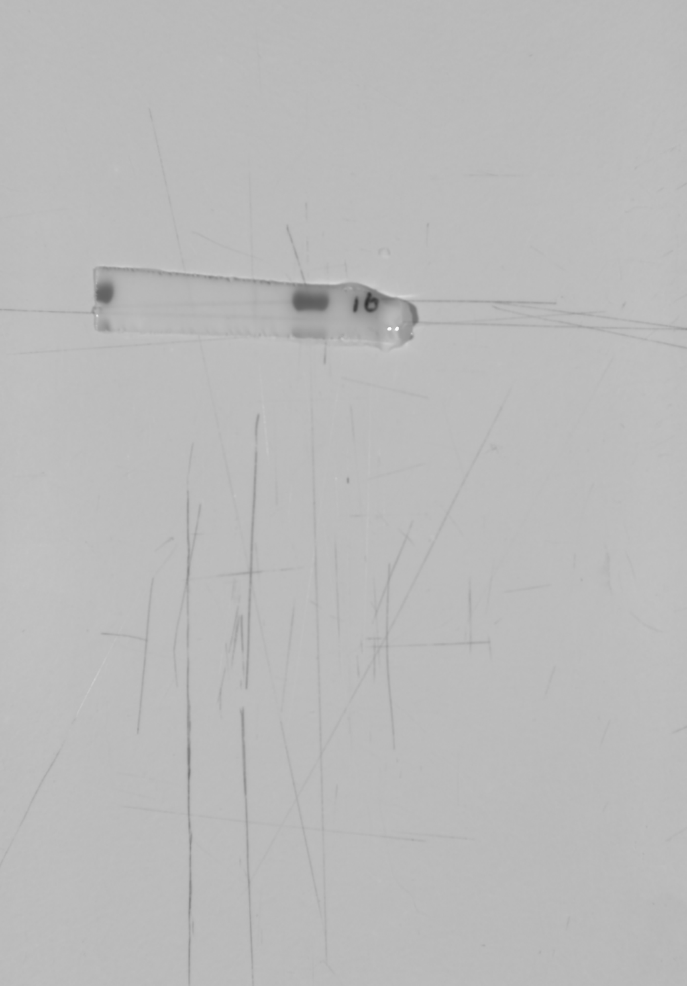


Beclin-1


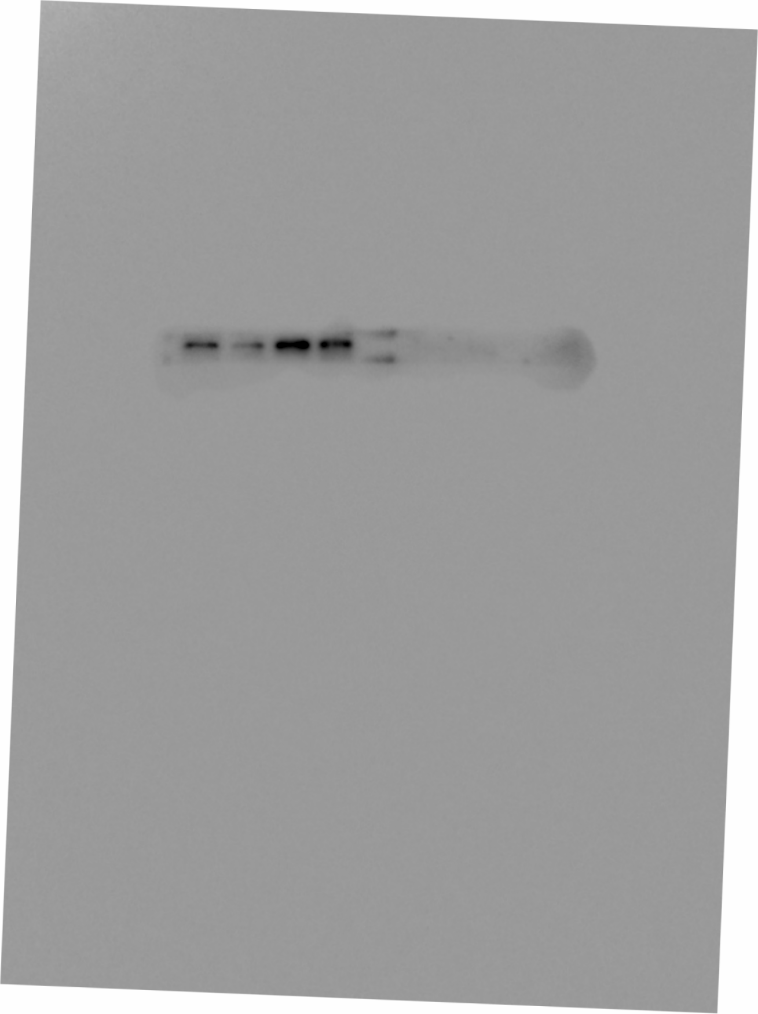


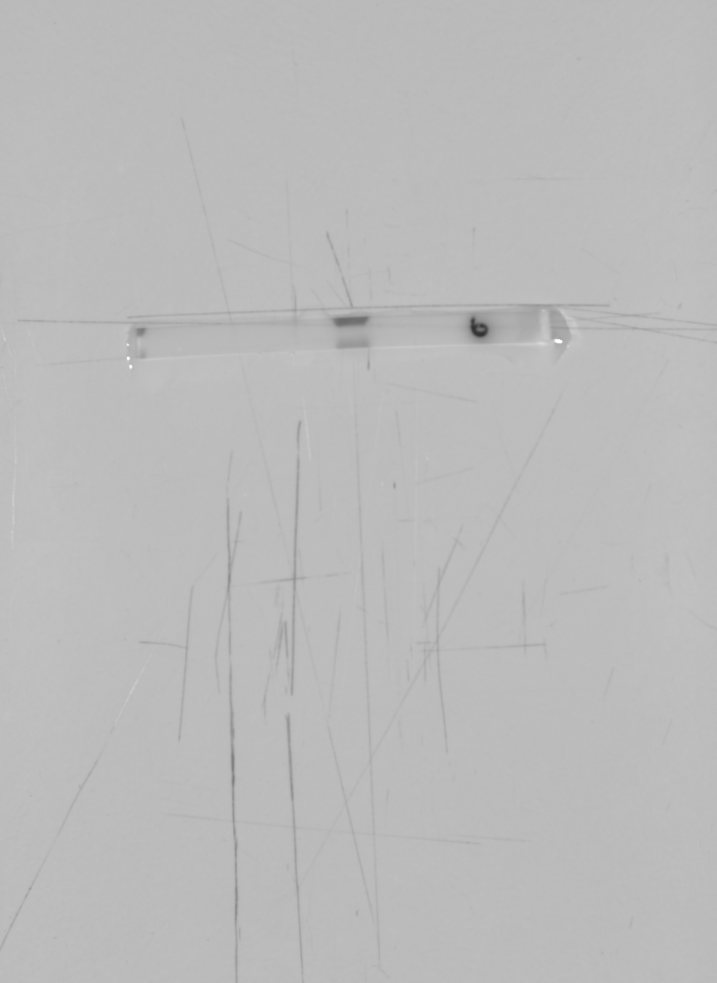


GAPDH


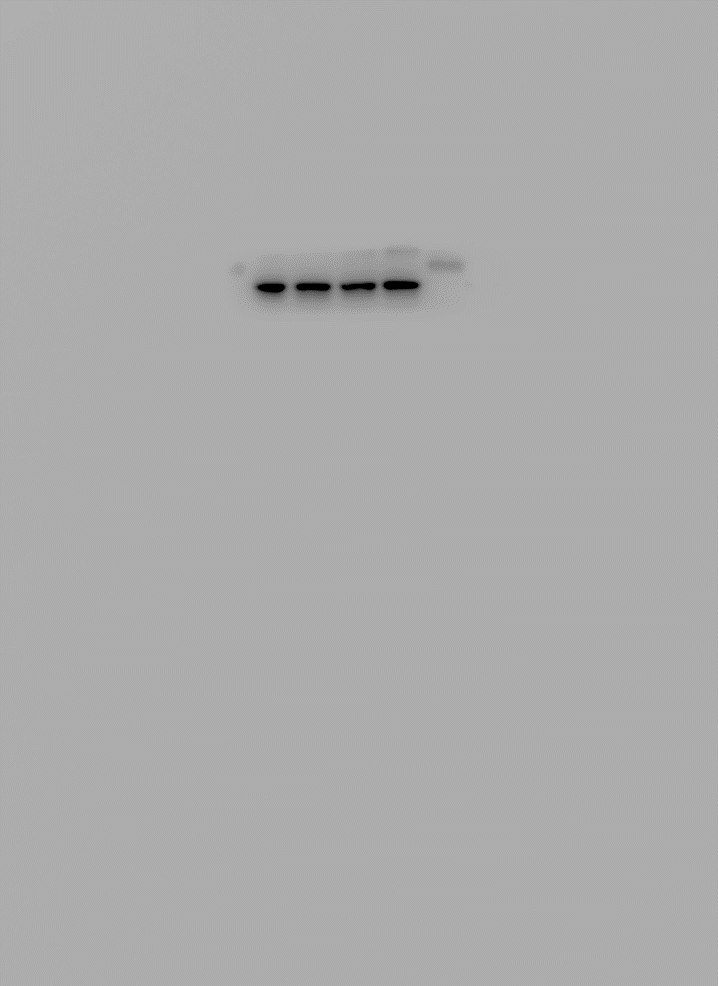


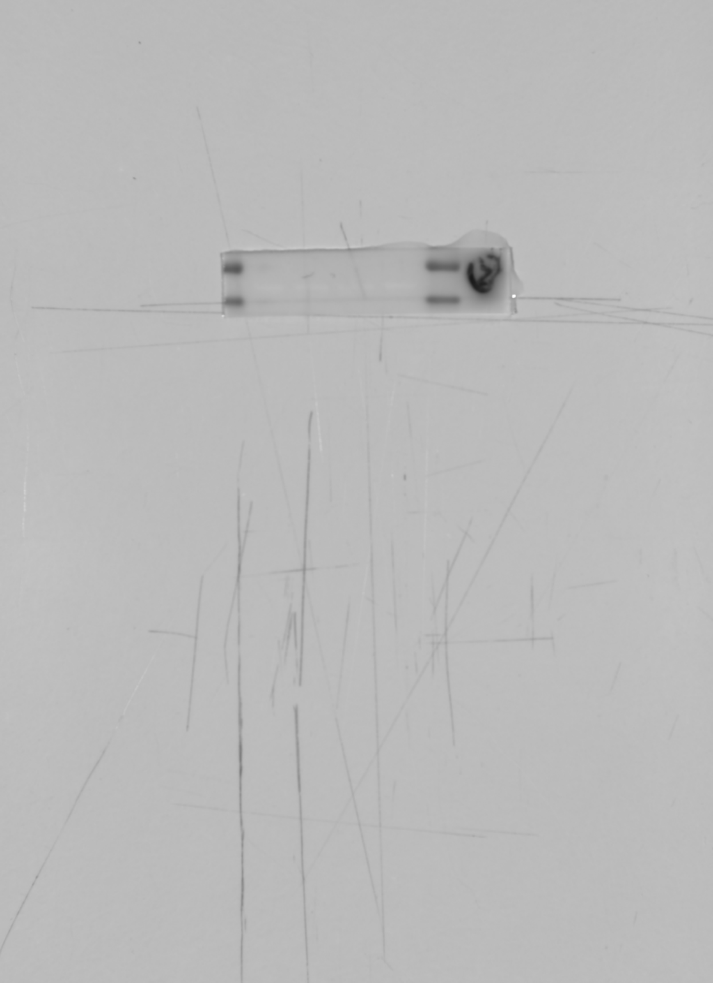


Fig4

p-p53


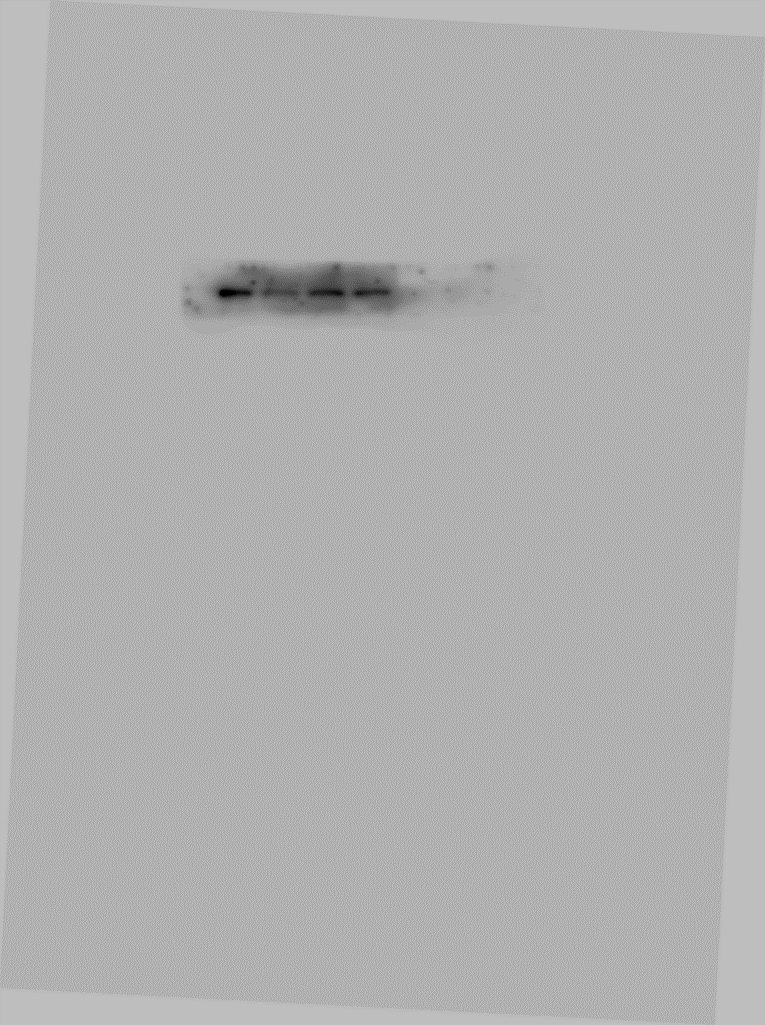


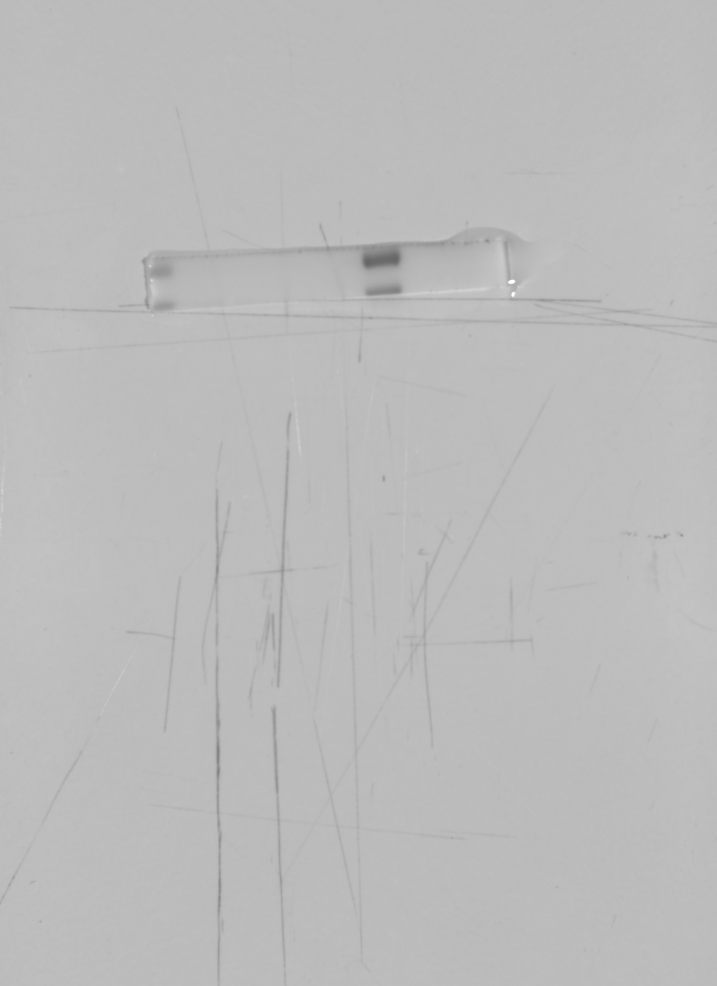


p53


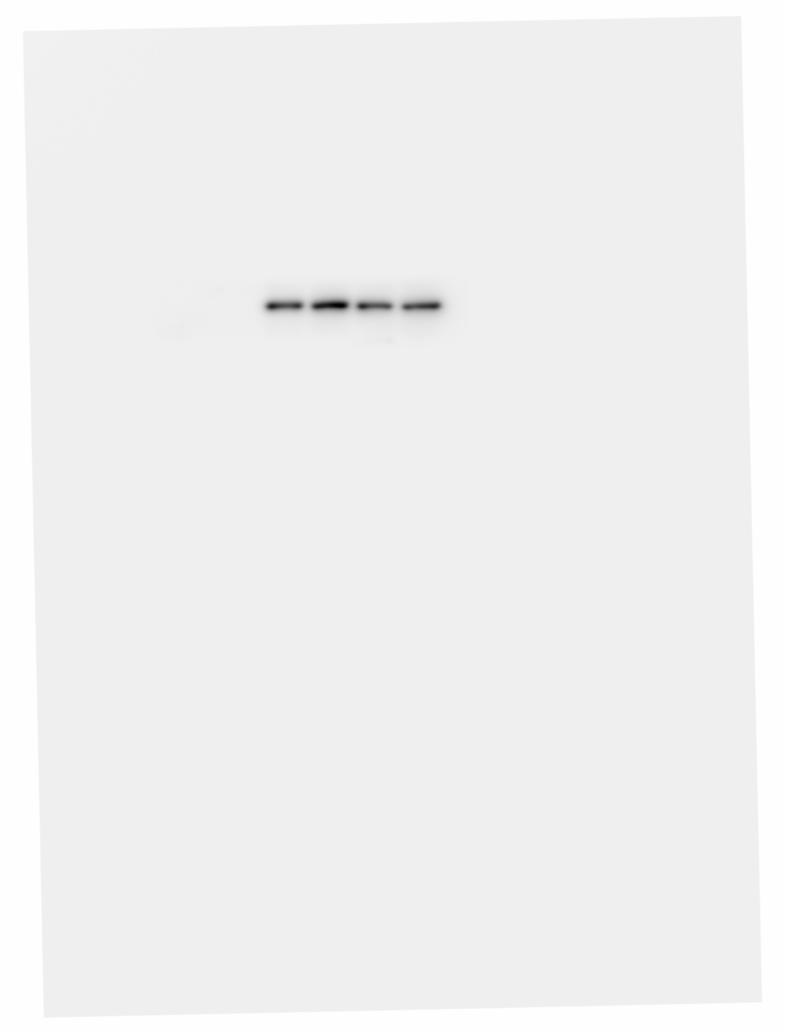


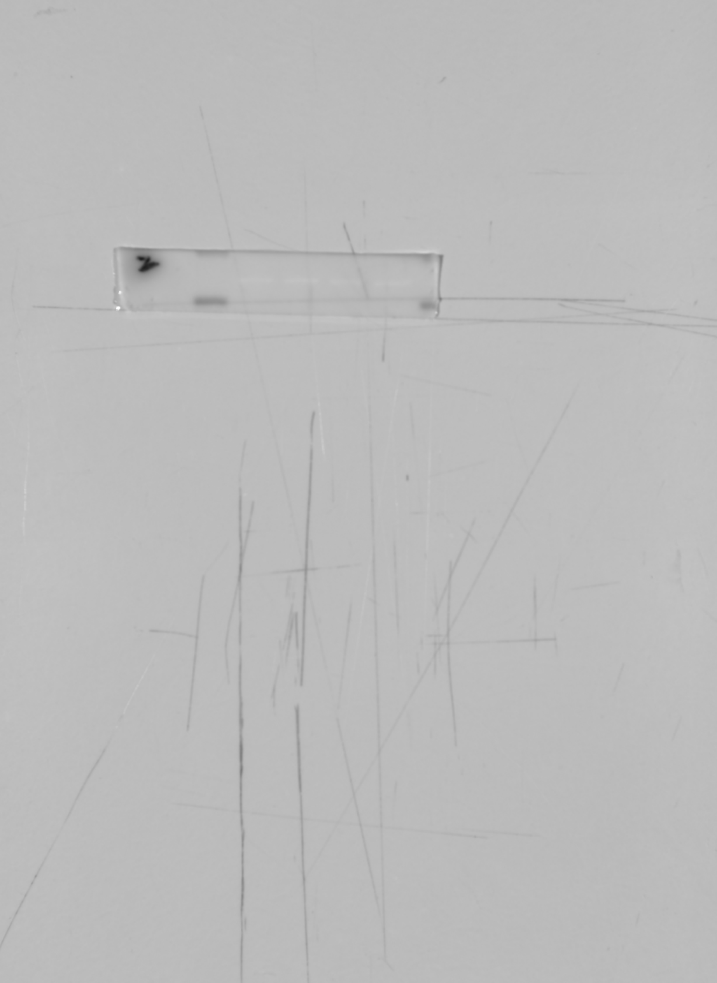


GAPDH


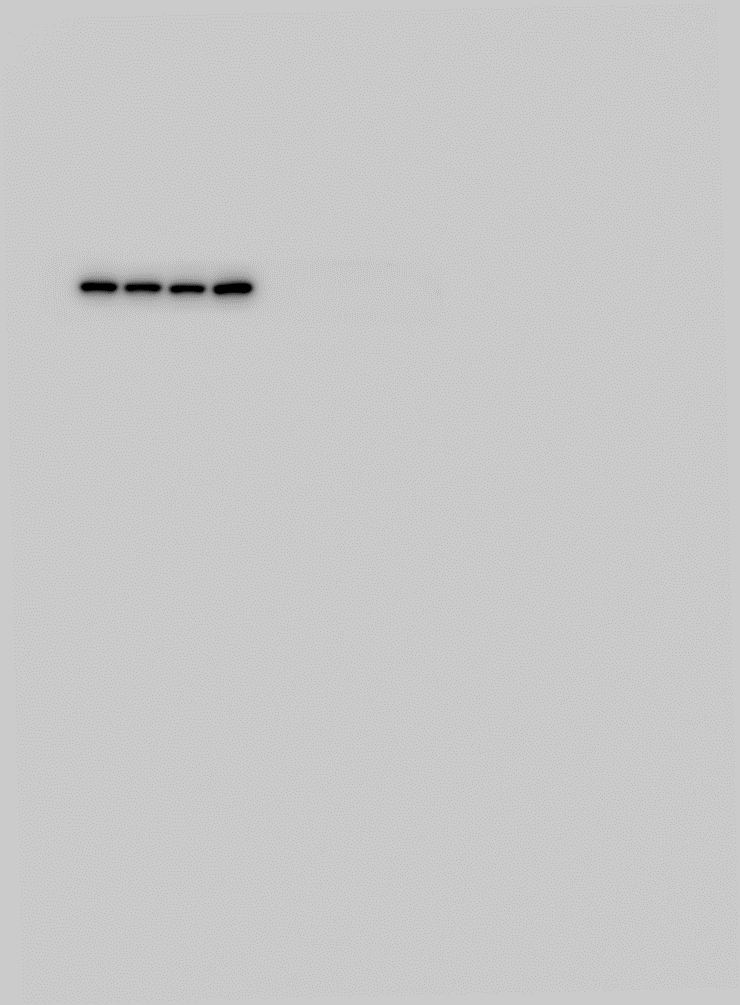


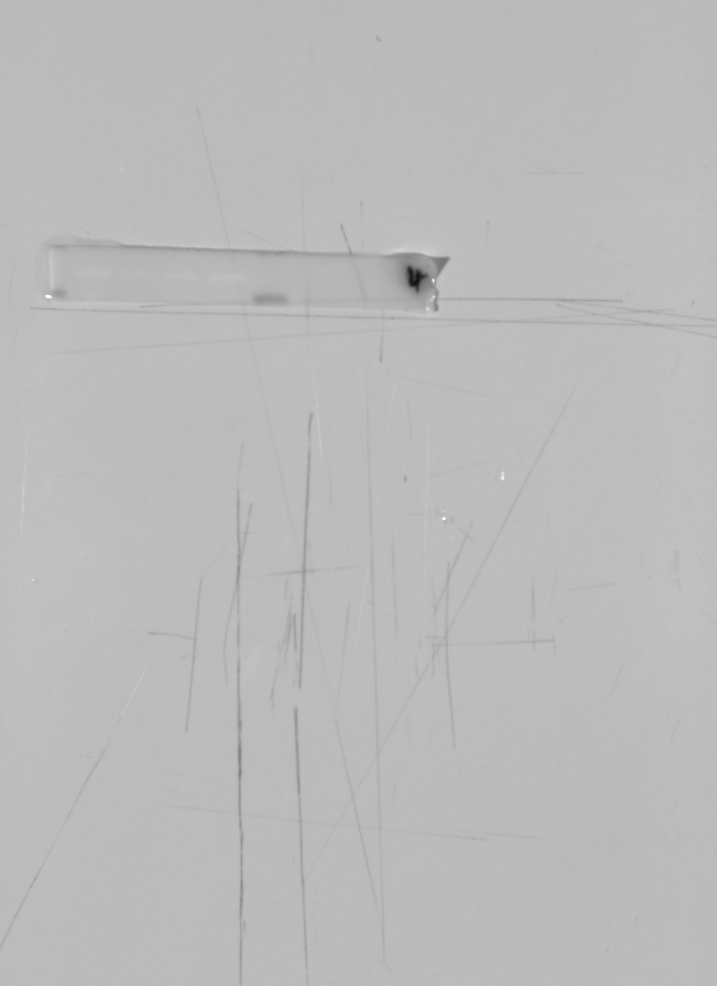


p-mTOR


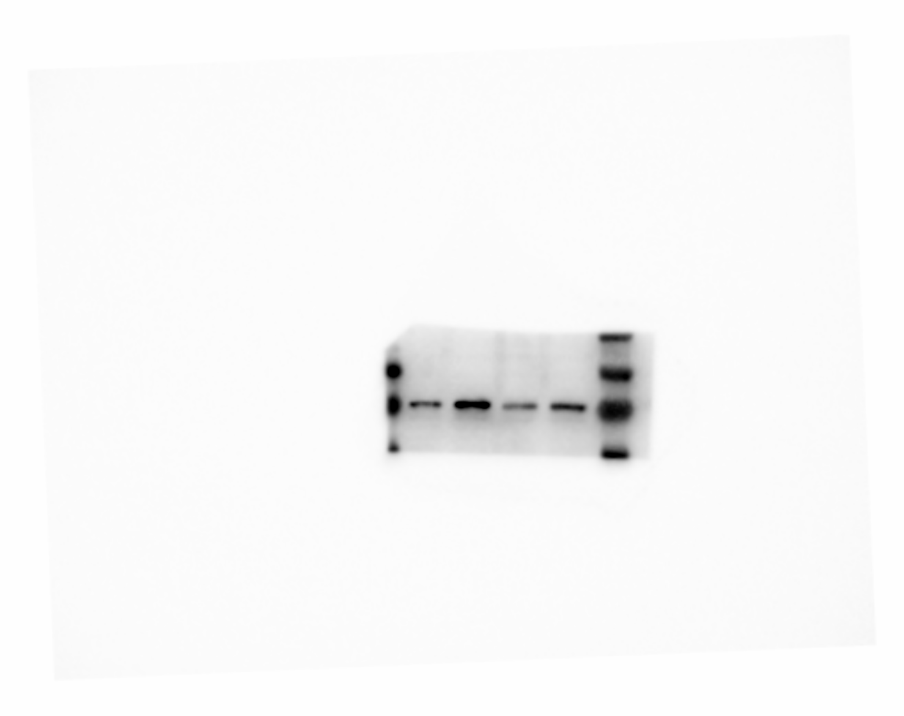


mTOR


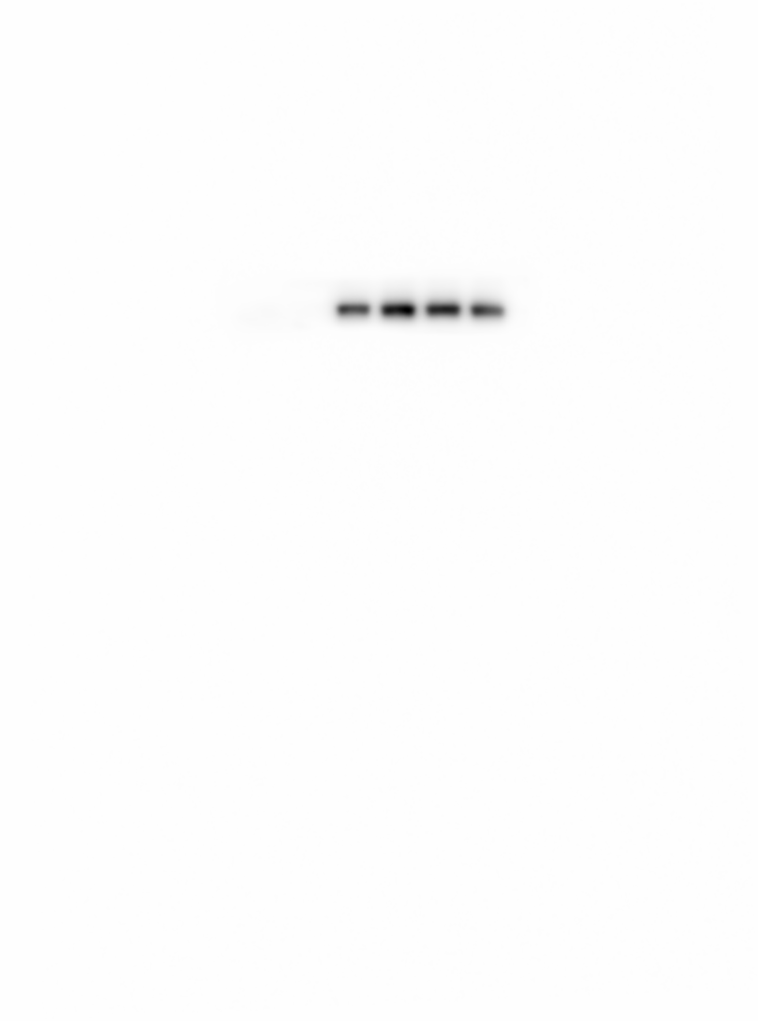


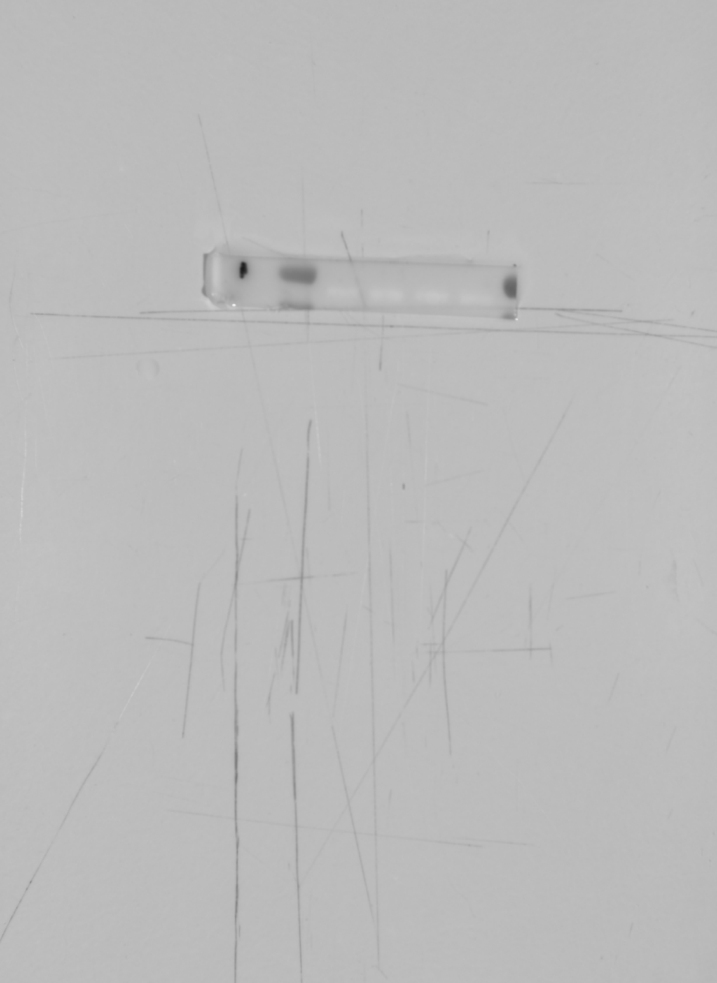


GAPDH


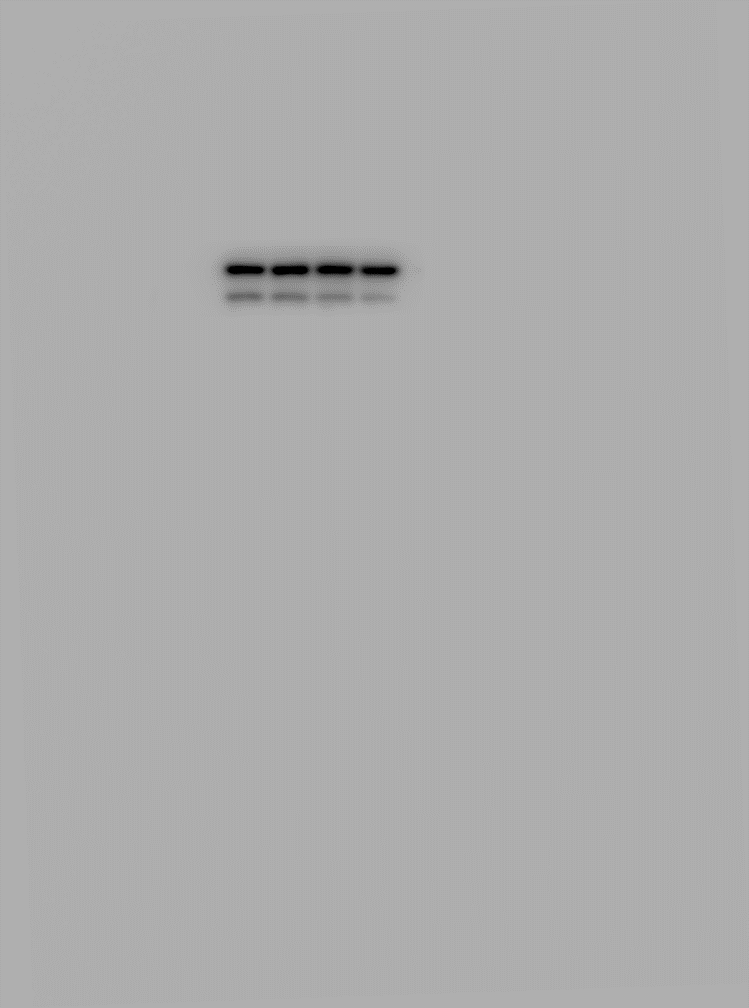


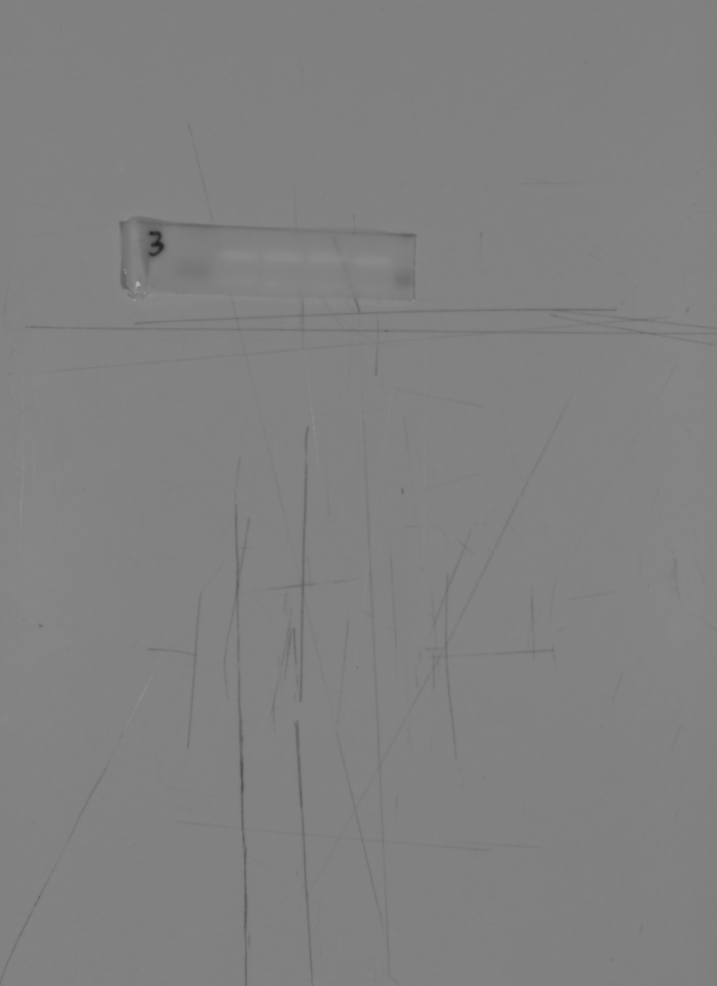


Fig7

LC3B


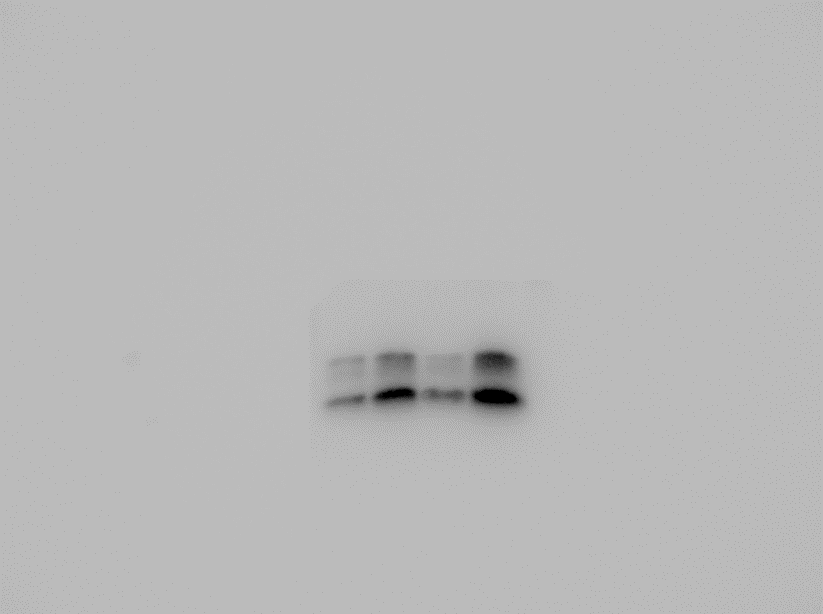


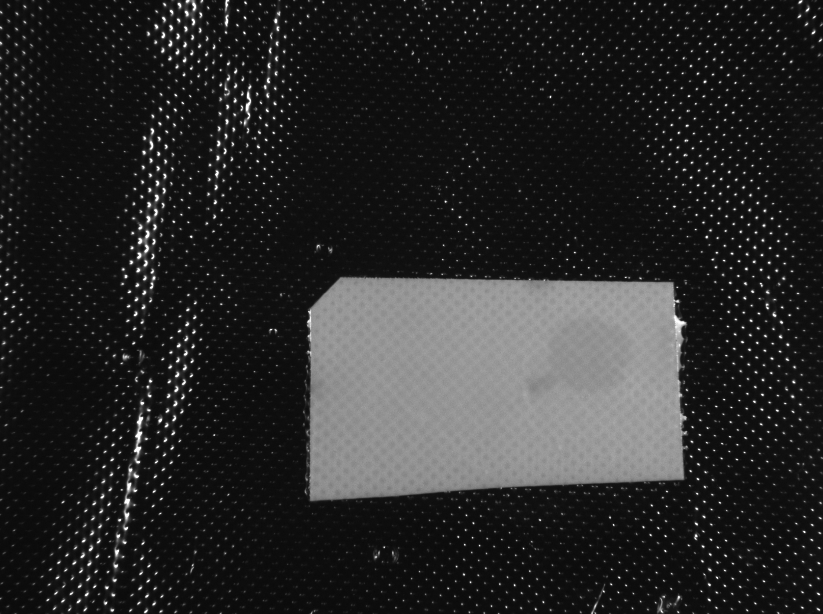


Beclin-1


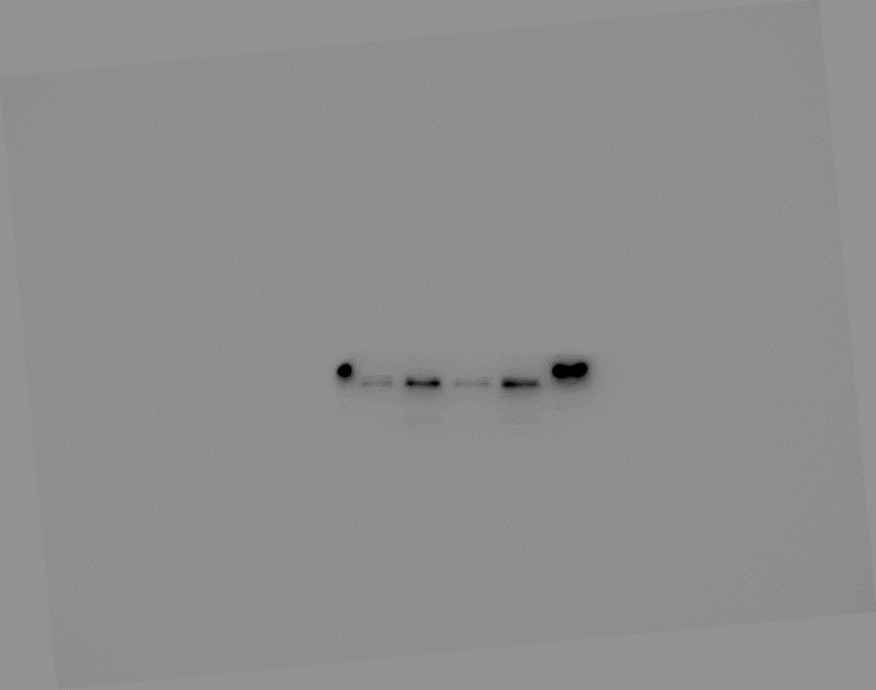


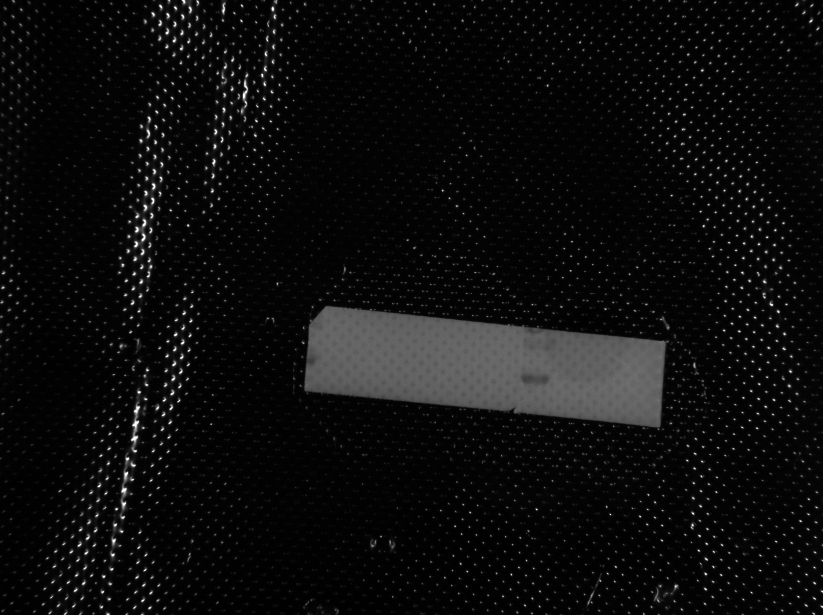


GAPDH


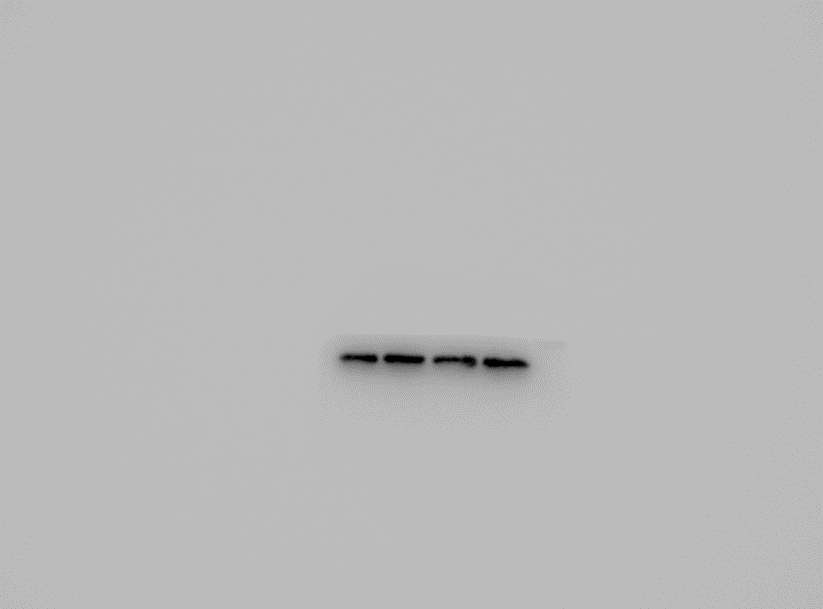


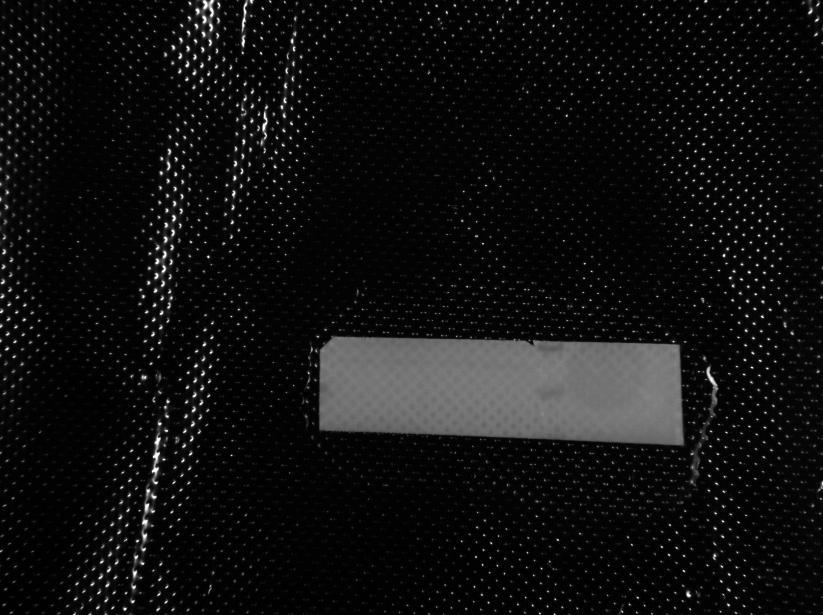


Fig8

p-p53


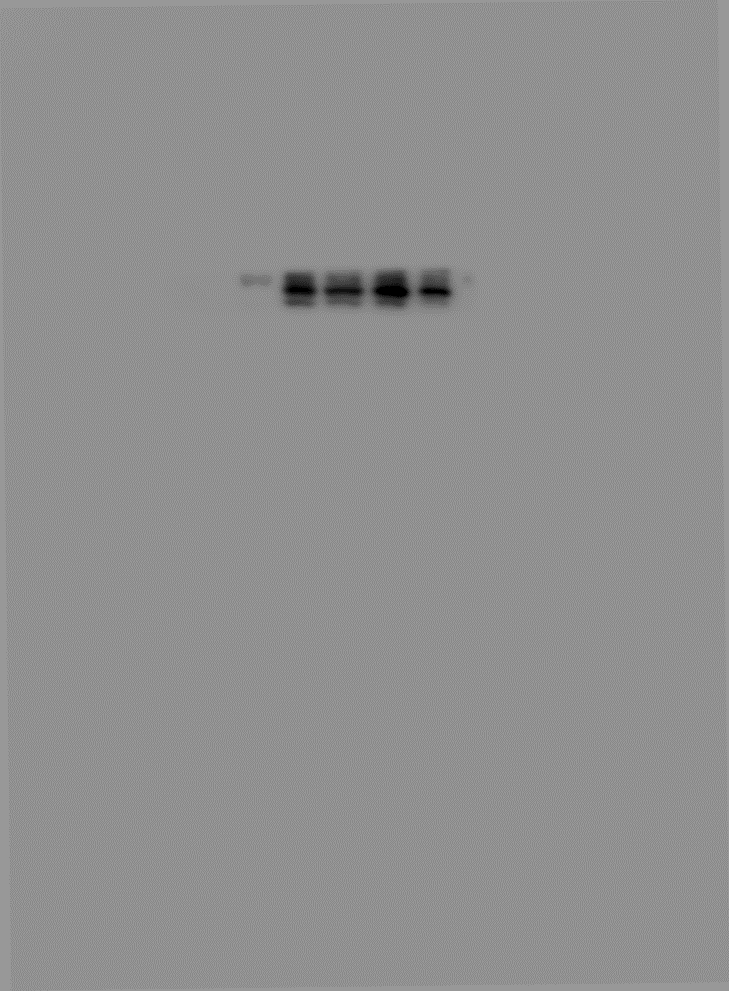


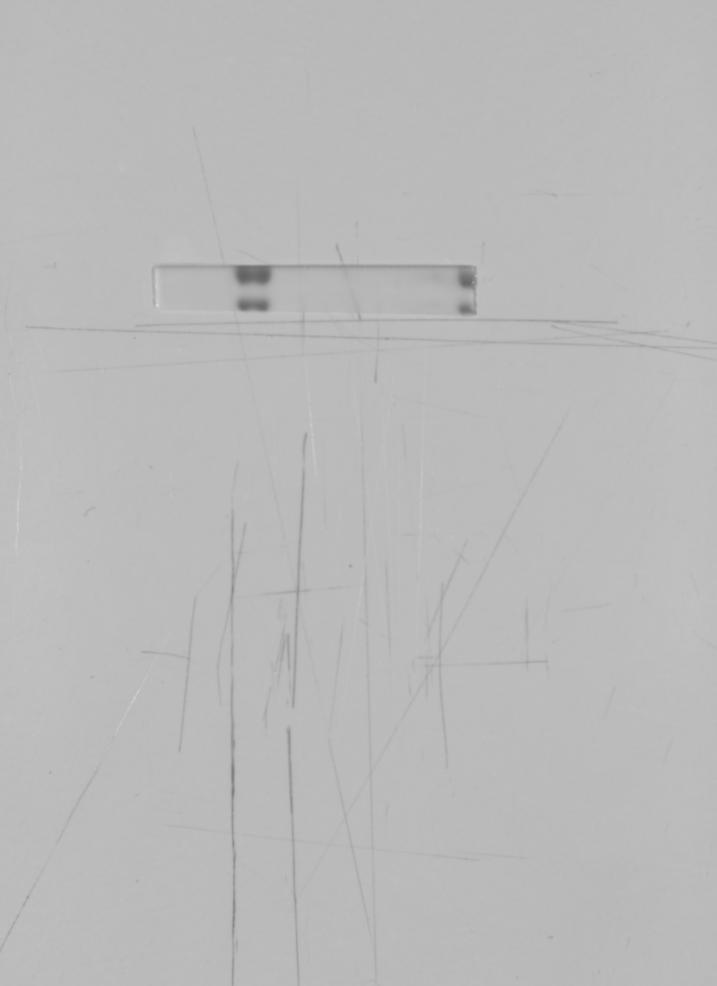


P53


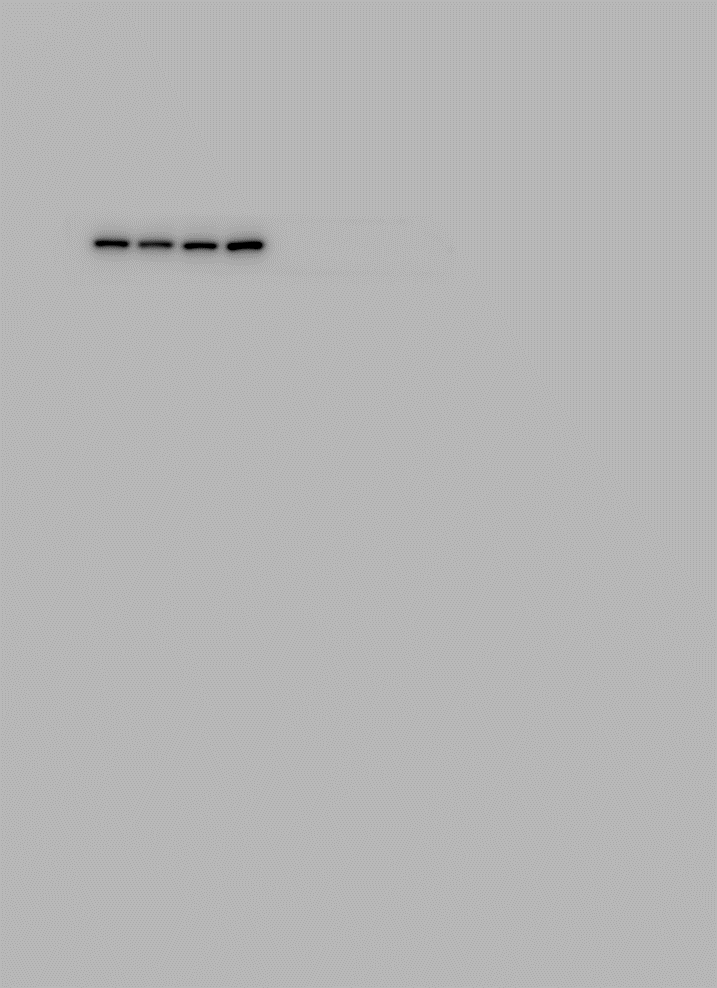


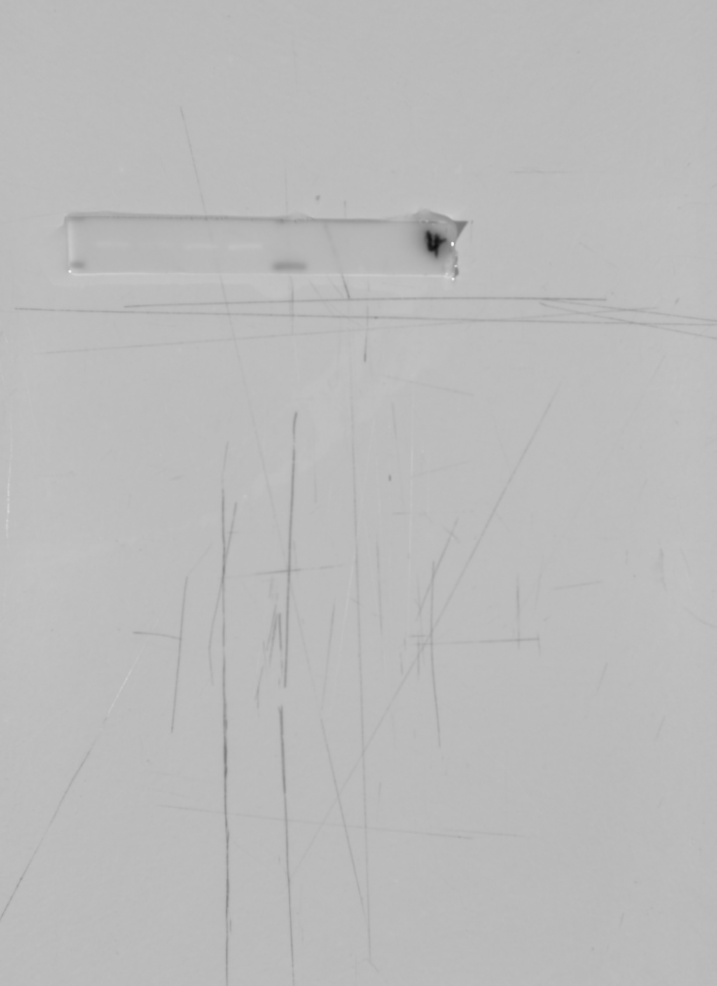


GAPDH


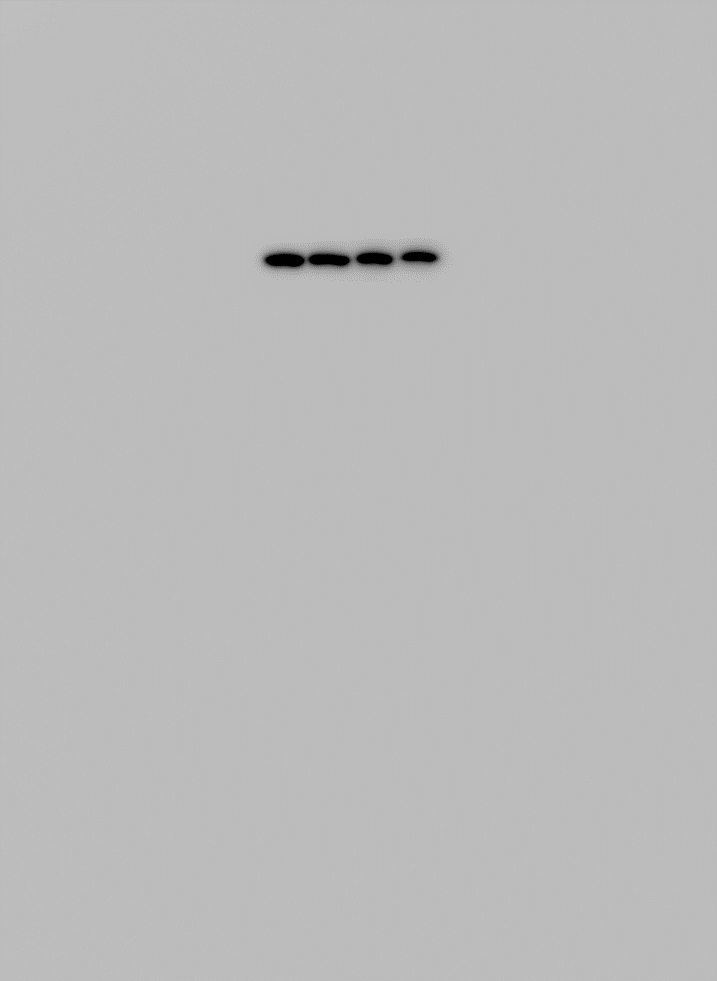


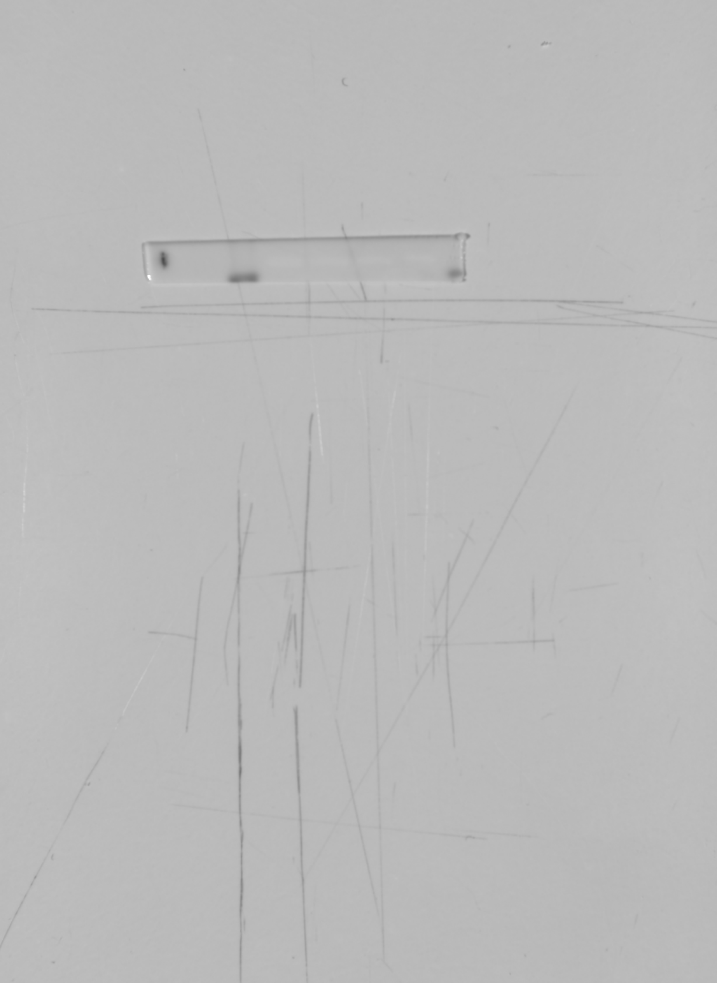


p-mTOR


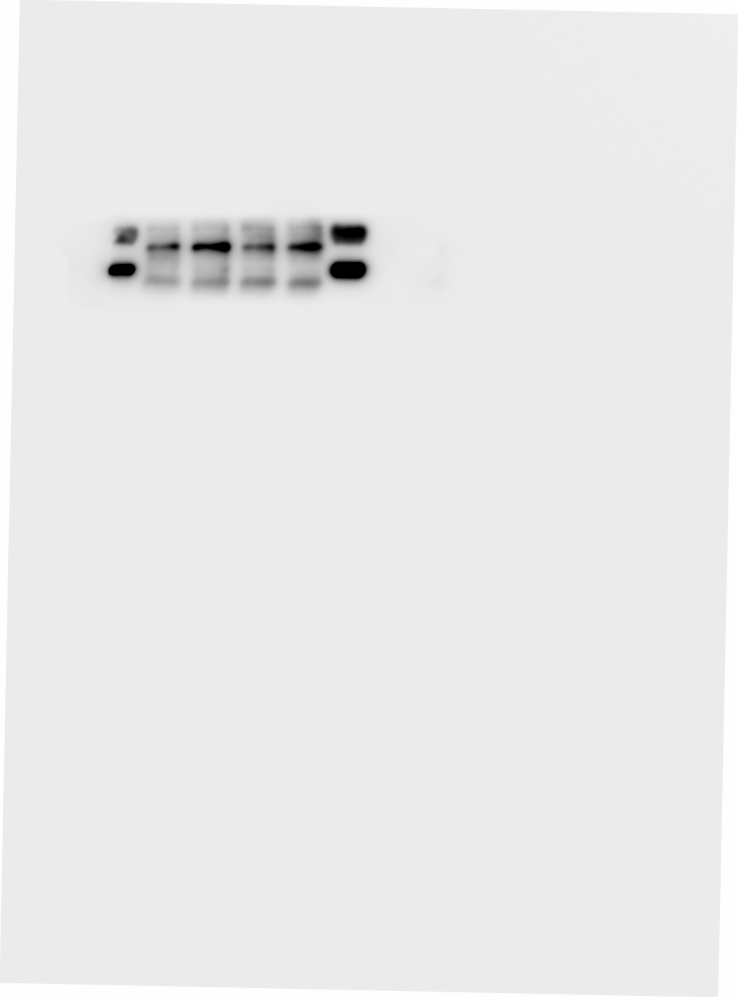


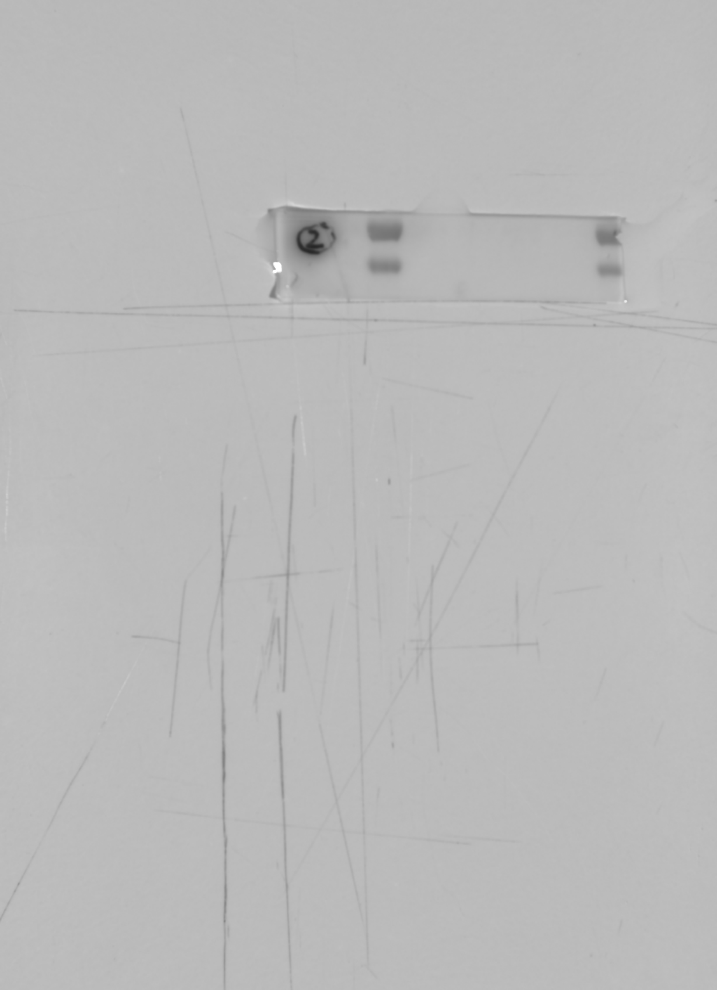


mTOR


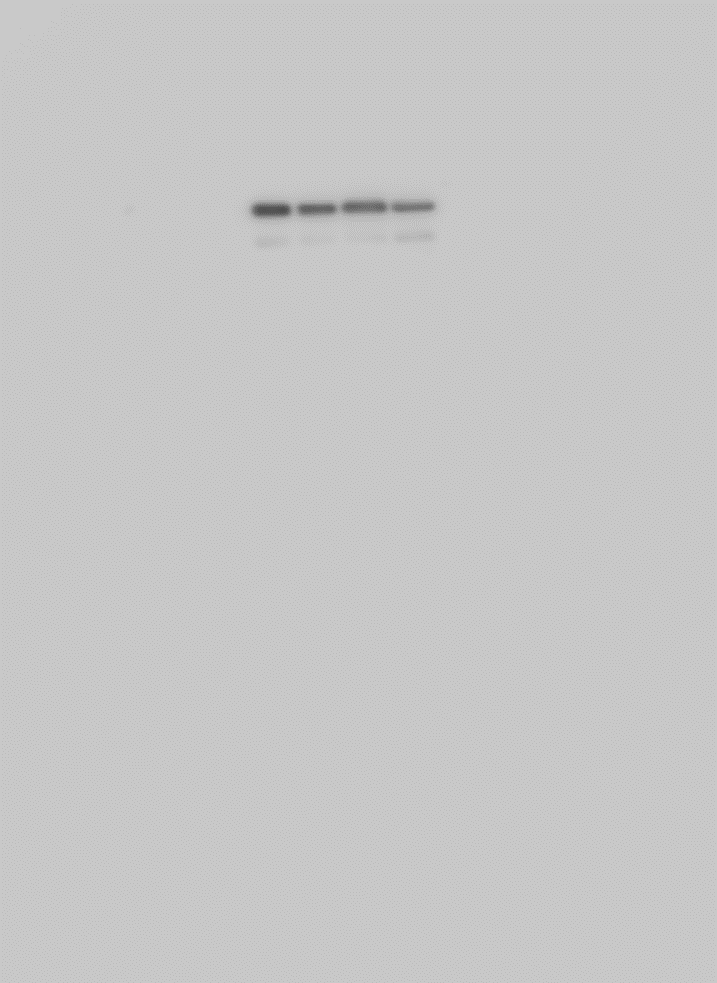


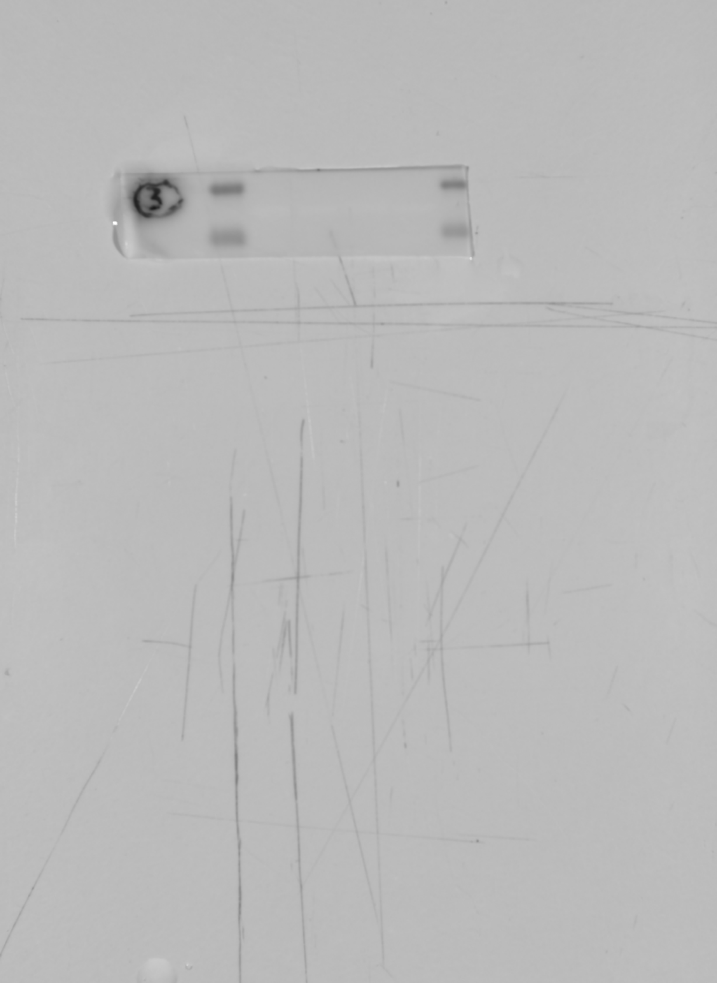


GAPDH


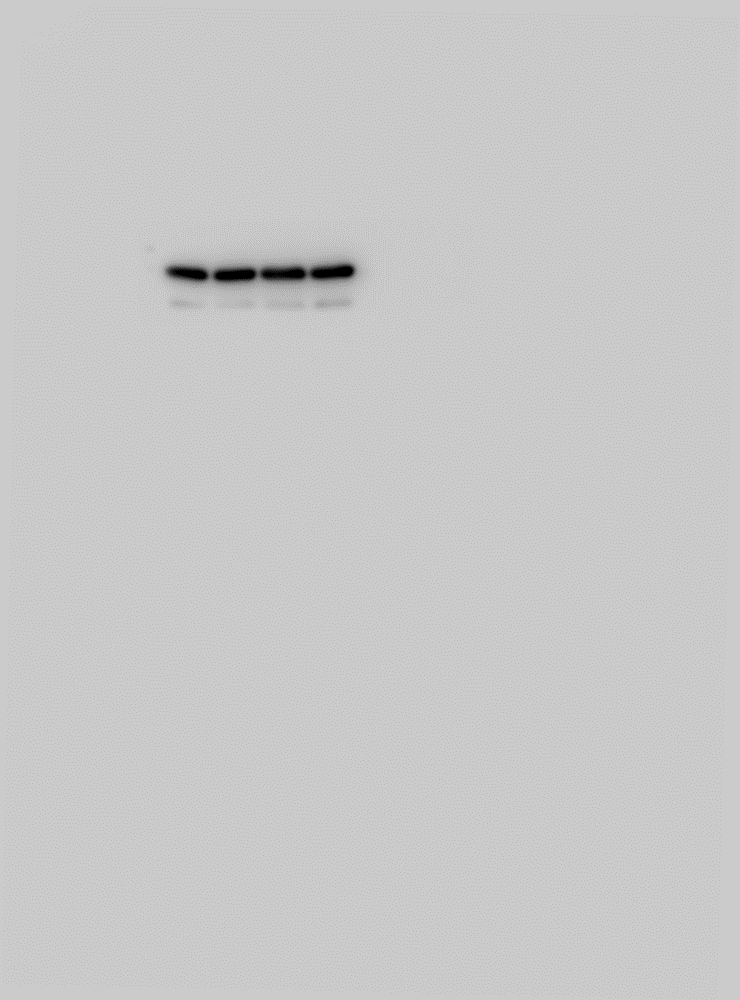


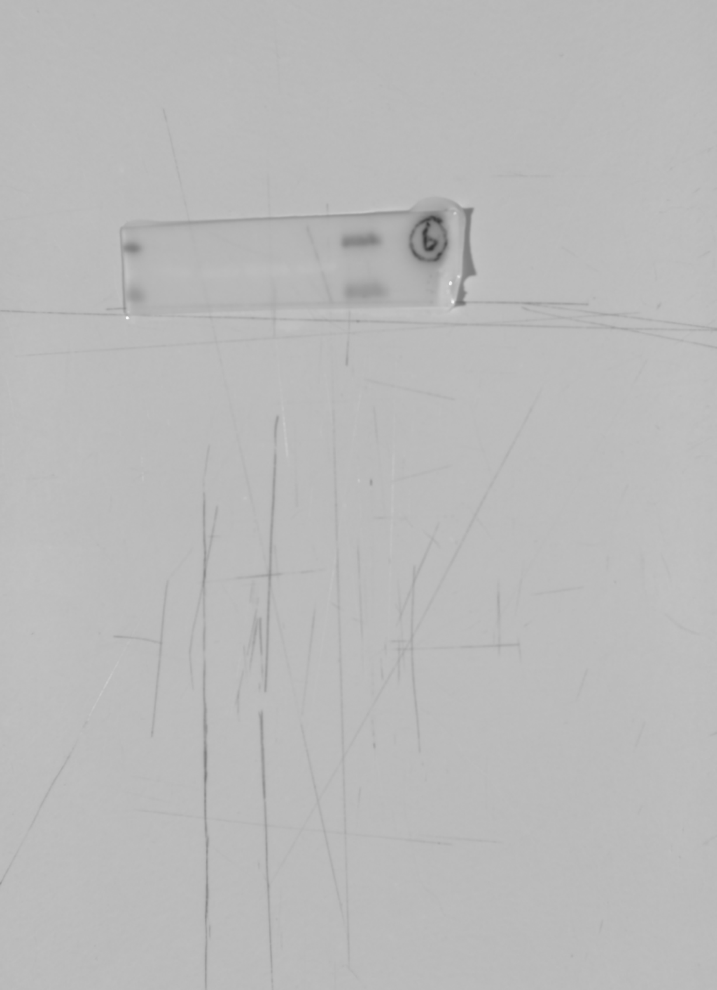

Supplement: Supplementary file 1 [file DataSheet1.ZIP › original gels.docx]
